# Plant metacaspases orchestrate wound‐induced pathways for immunity and tissue regeneration

**DOI:** 10.1111/tpj.70531

**Published:** 2025-11-19

**Authors:** Zhili Pang, Haijiao Liu, Qun Liu, Eric Lam

**Affiliations:** ^1^ Department of Plant Biology Rutgers the State University of New Jersey New Brunswick New Jersey 08901 USA; ^2^ Department of Materials Sciences and Chemical Engineering Stony Brook University New York 11794 USA; ^3^ Biology Department Brookhaven National Laboratory Upton New York 11973 USA

**Keywords:** wounding response, metacaspases, phytocytokines, DAMP, immunity, regeneration, *At*MC4, *At*MC9, WOX5

## Abstract

Wounding in plants elicits immunity and tissue repair, but how these responses are coordinated has yet to be elucidated. While plant metacaspases resemble animal caspases in structure and immunity induction, their role in tissue repair and regeneration is unknown. Using Arabidopsis mutants lacking type II metacaspases *At*MC4 or *At*MC9, we found that the majority of the highly induced, wound‐responsive genes in *Arabidopsis thaliana* are suppressed by the loss of *At*MC4, while *At*MC9 plays an auxiliary role in defense activation. Specifically, *At*MC4, but not *At*MC9, is required for the activation of genes involved in tissue repair, such as the developmental regulator *WOX5*, as well as for root regeneration from excised leaves. Instead, *At*MC9 mediates the repression of a subset of basal immunity genes, which modifies the wound‐activated defense response from that induced by molecular patterns such as the bacterial flg22 elicitor. Our results thus reveal a conserved protease module that coordinates plant defense and tissue repair upon wounding. They could be new targets to improve crop performance and plant transformation protocols that involve tissue wounding before transgenic plant selection and regeneration. The groups of genes with distinctive requirements for the two metacaspases could provide markers to dissect how these specialized proteases affect different response pathways that underpin the multifaceted wounding response.

## Significance Statement

In addition to its known involvement in activating immunity functions through the maturation of the phytocytokine Pep1, the calcium‐dependent type II metacaspase *At*MC4 is found to be critical for the majority of wound‐induced transcription changes, including genes involved in tissue regeneration such as the key developmental regulator *WOX5*. The complex effects on diverse groups of wound‐inducible genes by *At*MC4 indicate the involvement of cryptic proteolytic targets that mediate its signaling functions.

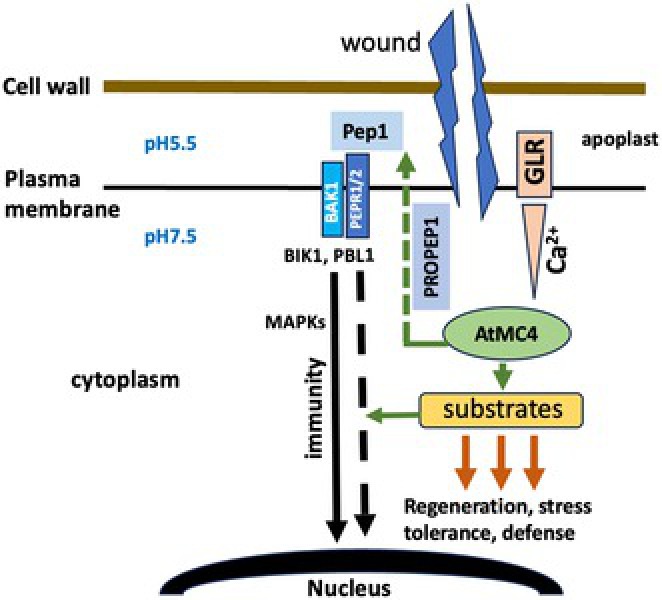

## INTRODUCTION

As sessile organisms, it is essential for plants to constantly survey their environment and make the necessary changes in their growth and development to maintain homeostasis and optimize fitness. In addition to pathogenic microbes, plants are constantly challenged by herbivorous predators and by mechanical damage from environmental factors. These physical damages, commonly called wounding, are manifested as disruptions in the integrity of the plasma membrane and the plant cell wall (Engelsdorf et al., 2018; Hoermayer & Friml, 2019). To recover from wounding, plants activate appropriate defense pathways to block opportunistic pathogens while initiating healing processes to regenerate healthy cells, tissues, or even new organs to replace those that were damaged or lost. Over the past 20 years, steady advances have been made in revealing the complex plant pathways that are induced during various types of physical injuries. These studies focus on two distinct responses: one is directed at understanding the cellular and molecular events that are necessary to drive regeneration and wound healing (Hoermayer & Friml, 2019; Ikeuchi et al., 2020), while the other addresses how the induction of basal immunity pathways occurs through the perception of two types of damage/danger‐associated molecular patterns (DAMPs), generated by the plant's own components via highly conserved signaling pathways involving a collection of receptor‐like kinases (Bartels et al., 2013; Ge et al., 2022; Yamada et al., 2016). From damaged cells, common cellular components, such as nucleotides, cell wall fragments, and amino acids (called cDAMPs for constitutive DAMPs) can leak into the apoplastic space between the plant cell wall and plasma membrane, and act cell‐autonomously as ligands that bind to specific transmembrane receptor kinases which then activate downstream immune signaling both locally and systemically (Ge et al., 2022). In addition, inducible DAMPs (iDAMPs) called phytocytokines can be produced upon perception of cellular damage to activate specific secondary signals that can stimulate basal immunity as well as tolerance to various abiotic stresses (Bartels & Boller, 2015; Nakaminami et al., 2018; Rhodes et al., 2021).

In an increasing number of reported cases, these phytocytokines are found to be propeptides that require proteolytic cleavage by specific convertases—such as metacaspases (MCs) or subtilases—for their conversion into mature peptides (Bartels & Boller, 2015; Ge et al., 2022; Rhodes et al., 2021). While calcium mobilization and reactive oxygen species (ROS) signaling are known to be early cellular responses to wounding (Hernandez‐Coronado et al., 2022), how iDAMP‐triggered immunity can be coordinated with the tissue regeneration process is not well understood (Hoermayer & Friml, 2019). This is especially important to resolve since the induction of strong plant defenses is known to antagonize plant growth and development (Hernandez‐Coronado et al., 2022; Huot et al., 2014; Major & Campos, 2022). Recent advances in understanding the precise mechanisms that regulate convertases, specifically the conserved Arabidopsis *At*MC9 and *At*MC4 proteases, which are required to produce the cell death‐inducing peptide Grim Reaper (GRIp) upon oxidative stresses and a phytocytokine Pep1 upon wounding (Hander et al., 2019; Wrzaczek et al., 2015), respectively, prompted us to examine how these tightly controlled proteases may help to orchestrate the global transcriptional landscape invoked upon wounding. Here, using comparative transcriptomics, we studied *Arabidopsis* genotypes with defective *AtMC4* or *AtMC9* genes to reveal their roles in wound response. We found that *At*MC4 is essential for initiating a strong transcriptional response, including a key developmental gene *WOX5* (Pi et al., 2015), upon wounding. In contrast, *At*MC9 assists in the activation of a subset of *At*MC4‐dependent defense genes and modifies the immune response to Pep1 by repressing specific genes during wounding. Both MC‐encoding genes exhibit unique as well as overlapping functions to coordinate defense activation and tissue regeneration while modulating the basal immune response to minimize interference with growth and development (Hernandez‐Coronado et al., 2022; Huot et al., 2014; Major & Campos, 2022).

## RESULTS

### Metacaspase functions and substrate specificities

In *Arabidopsis thaliana*, six type II metacaspase (MC) genes encode enzymes involved in stress responses and development. *At*MC4 and *At*MC9 are constitutively expressed members, unlike *At*MC5‐8, which show low expression under ambient conditions (Lam & Zhang, 2012; Tsiatsiani et al., 2013). These MCs are synthesized as inactive zymogens, which are activated under appropriate conditions by autolytic cleavage at a conserved site in the linker between the p20 and p10 domains of these proteins (Lam & Zhang, 2012). *At*MC4 to *At*MC8 require millimolar concentrations of calcium for optimal enzyme activation, while *At*MC9 is activated only by acidic pH (Fortin & Lam, 2018; Vercammen et al., 2004). *At*MC4 enhances immunity and induces cell death in response to biotrophic bacterial pathogens (Watanabe & Lam, 2011a), while *At*MC9 facilitates xylem formation by *postmortem* clearance of the dead cell content (Bollhöner et al., 2013). Both MCs are known to be required for maturation of phytocytokines including GRIp and Pep1, the latter being one of the eight Propeps involved in pathogen and herbivore resistance (Bartels et al., 2013; Hander et al., 2019; Wrzaczek et al., 2015). A report that genetically linked *At*MC4 to Propep1 processing upon wounding in Arabidopsis demonstrated that while this convertase activity is lost in the *atmc4‐1* background in leaf tissues, it remains active in the root tissues (Hander et al., 2019). This indicates that a different type II MC in addition to *At*MC4 may be capable of processing Propep1 in the root.

**Figure 1:**
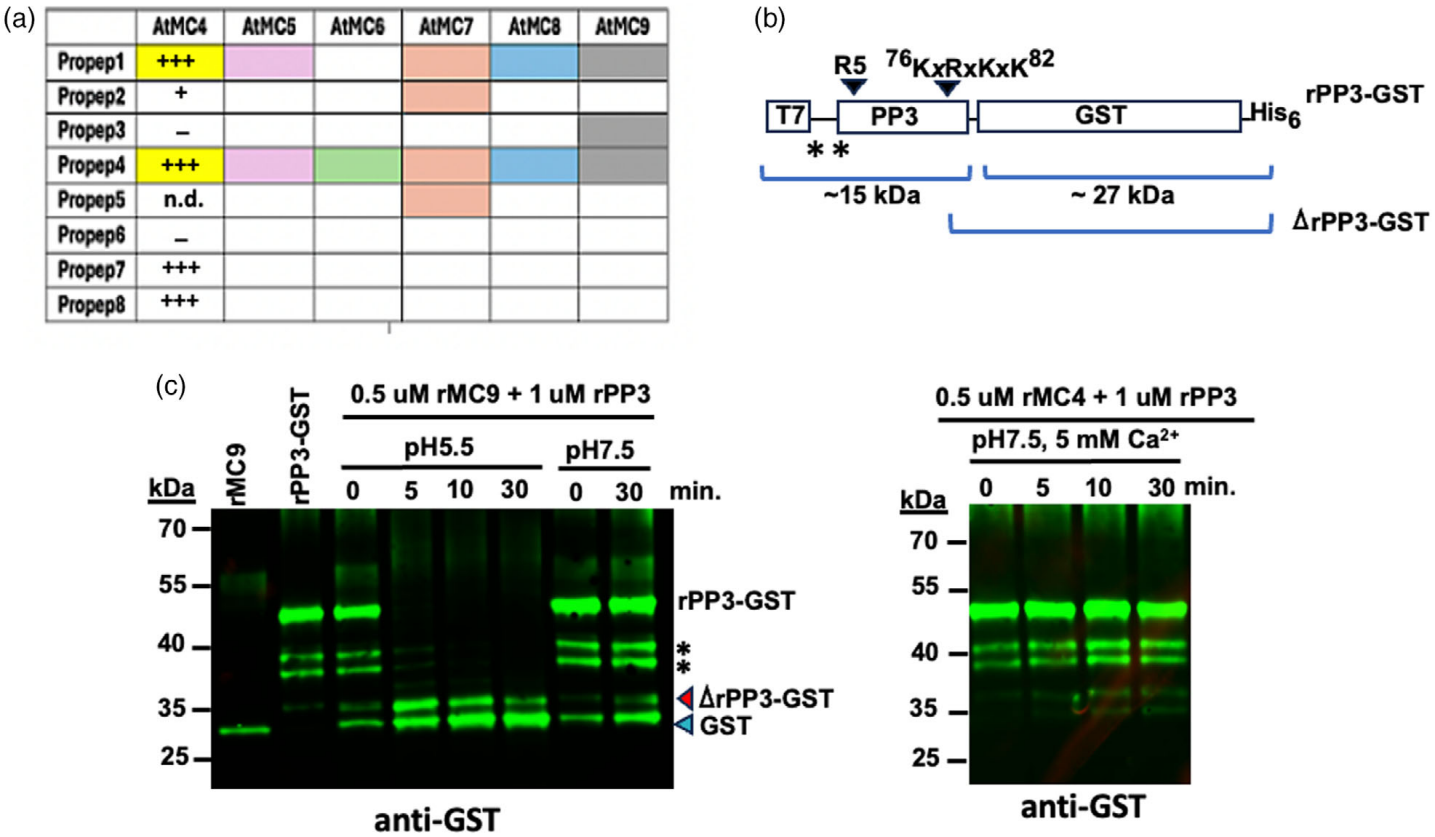
Differential Propep specificity for members in the Arabidopsis type II metacaspase family. (a) Using AlphaFold2, each of the six members (*At*MC4–*At*MC9) in the Arabidopsis type II metacaspase family were used to query potential cleavage of the eight Propep (PP) sequences. Predicted targets are labeled with colored boxes for each metacaspase. The *in vitro* processing results with recombinant *At*MC4 and a comparable C‐terminal glutathione S‐transferase (GST) fusion for each PP sequence from Hander et al. (2019) are shown for comparison. The results for the relative rate of processing each PP are shown by the number of “+” sign. “n.d.”: not determined. (b) Structure of Propep3 (PP3) sequence in fusion with GST‐tag in p*ET‐23a*(+). The positions of potential cleavage sites were labeled with black arrowheads. Size of the boxes are not to scale. Asterisks under the linker region between the T7 antigen site and the N‐terminal end of the PP3 sequence indicate potential alternate translation start sites. (c) Confirmation of the predicted cleavage of PP3 by *At*MC9 (MC9), but not *At*MC4 (MC4). PP3 was produced as a fusion to the (GST) protein to facilitate its tracking via western blot analysis with an anti‐GST antibody. Asterisks indicate potential products with alternate translation start sites. Red arrowhead indicates the cleavage product with PP3 C‐terminal end fragment fused to GST. Blue arrowhead indicates a background band that cross‐reacted with the anti‐GST antibodies from the recombinant *At*MC9 protein purified from bacteria with a similar size as the GST protein alone. No cross‐reacting band was observed with the rMC4 preparation. Site‐specific mutagenesis data demonstrate that the cluster of four basic amino acid residues from K76 to K82 in PP3, but not R5, likely contains the required cleavage site by MC9 to generate the Pep3 phytocytokine (see Figure S1 for details).

Using AlphaFold2 (Jumper et al., 2021), we predicted MC‐Propep interactions as shown in Figure 1a. Our approach predicts Propep4 is a universal substrate for all six type II MCs. On the other hand, Propep1 can be cleaved by most of the type II *AtMCs* tested, except for *At*MC6, while *At*MC7 appears to be specifically required for Propep2 and Propep5, and *At*MC9 is uniquely able to cleave Propep3. Propep6–8 showed no significant predicted interactions with the six type II MCs tested with our current informatic pipeline (see “Materials and Methods” section for details). Published work using *in vitro* assays with recombinant proteins confirmed *At*MC4 actively cleaves Propep1 and Propep4 but does not significantly cleave Propep2, Propep3, or Propep6 (Hander et al., 2019), aligning with our predictions (Figure 1a). However, r*At*MC4 was reported to actively cleave Propep7 and Propep8 in this previous work, thus contradicting predictions and indicating our current AlphaFold pipeline does have some limitations. Another published study using a protoplast transient expression protocol suggested that all type II MCs, including *At*MC6, could process Propep1, and that *At*MC4 cleaves most Propeps, except Propep6 (Shen et al., 2019), in conflict with our predictions and with the earlier data for Propep3 (Hander et al., 2019). Interestingly, our predicted cleavage of Propep1 by *At*MC9 is consistent with the protoplast transformation study. Indeed, our recent study of *At*MC9 structure and function demonstrated that despite its distinct dependence on acidic pH instead of calcium for activation, recombinant *At*MC9 is active in cleaving recombinant Propep1 *in vitro* (Liu et al., 2025).

To validate the other prediction, we compared the cleavage of recombinant Propep3 fusion protein by recombinant *At*MC4 and *At*MC9, under their respective conditions for activity (Figure 1c). As found previously (Hander et al., 2019), *At*MC4 failed to process Propep3‐GST fusion protein in the presence of calcium, while *At*MC9 could process this fusion protein at pH 5.5, but not at pH 7.5 (Figure 1c). As expected, the active site cysteine mutation C147G in *At*MC9 inactivated its ability to cleave Propep3‐GST, while cleavage of Propep3‐GST by *At*MC9 was not observed at pH 7.5 (Figure S1a,c; Figure 1c). In addition to using anti‐GST serum, we also confirmed our interpretation of the western blot data using anti‐T7 serum against the N‐terminus of the recombinant Propep3‐GST fusion protein (Figure S1c). Collectively, our data demonstrate that *At*MC4 and *At*MC9 have overlapping and distinct substrate specificities. Some of the conflicting results observed from previous protoplast‐based studies (Shen et al., 2019) might have resulted from the fact that protoplast preparation protocols are likely to create a wounded cell background that could complicate the interpretation of the results. In addition, activation of other endogenous MC‐encoding genes, such as *AtMC9*, or those encoding other plant proteases upon co‐transformation with the expression cassettes for Propep fusion genes and various metacaspases may further confound interpretation of the data.

### Identification of metacaspase‐dependent genes in the wounding response

Using comparative transcriptomics, we investigated how the loss of *At*MC4 and *At*MC9 affects the genome‐wide wounding response in *A. thaliana* leaves, following a leaf wounding protocol (Hander et al., 2019; Figure S2). Previous work showed *At*MC4 influences defense genes upon wounding (Hander et al., 2019) and exogenously applied Pep1 treatment alters the transcriptome with similar signatures to other MAMPs (Bjornson et al., 2021), but the broader roles of *AtMC4* and *AtMC9* remain unclear. For our present work, transcriptome analysis was conducted with *A. thaliana* leaves 4 h post‐wounding, a time point linked to peak initial transcription activation in several studies involving *Arabidopsis* leaf tissues (Hander et al., 2019; Ikeuchi et al., 2017).

**Figure 2:**
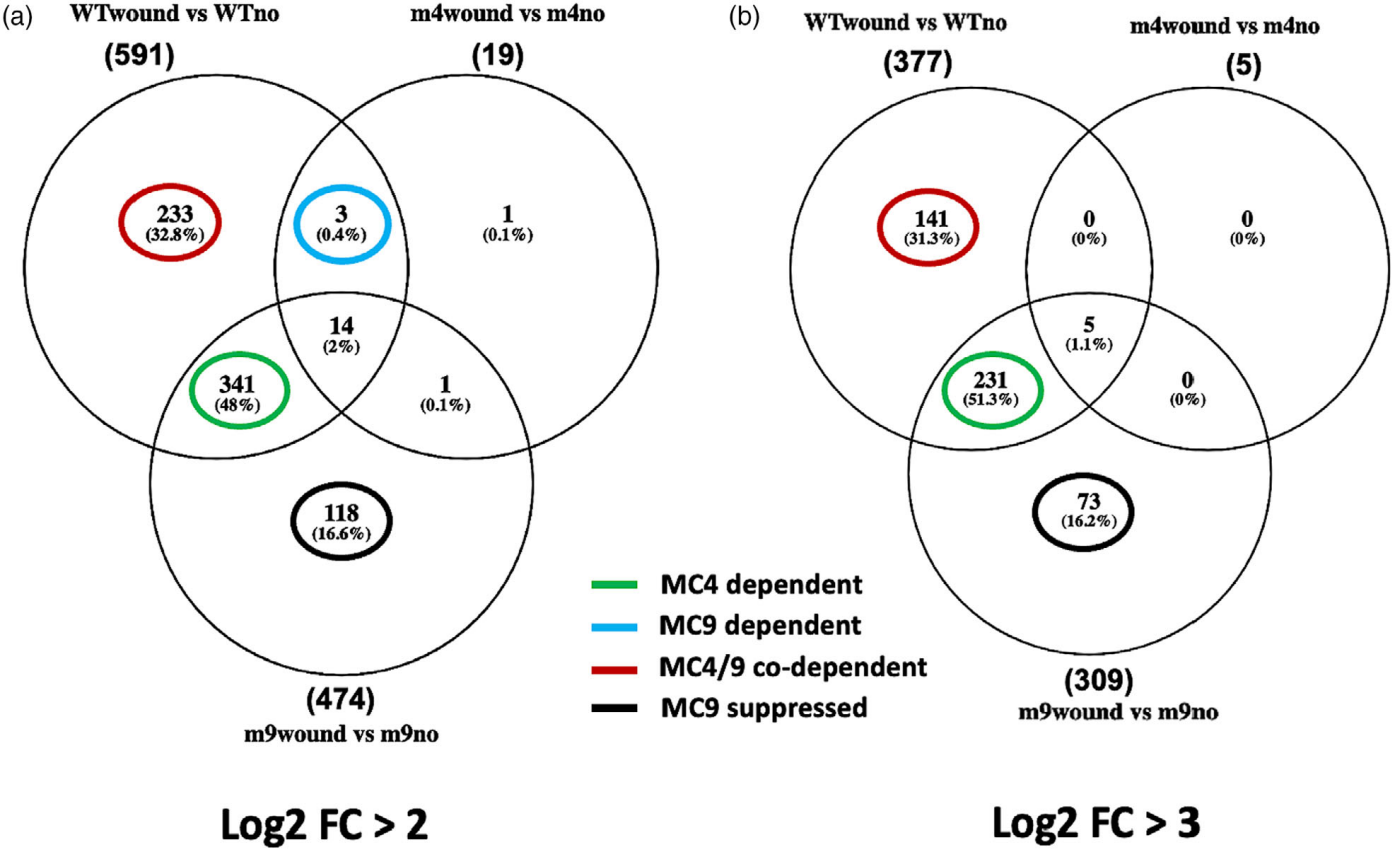
*At*MC4 mediates transcriptional responses to mechanical leaf wounding in *Arabidopsis*. (a) Differentially expressed genes (DEGs) with a Log2‐fold change (FC) >2. (b) DEGs with a Log2 FC >3. Transcriptomic results from the seedlings of WT (wild‐type), *atmc4‐1* (m4), and *atmc9‐1* (m9) genotypes were compared. “no”: no wounding controls. Total DEGs in each group are shown in parentheses.

Comparing wild‐type (WT), *atmc4‐1*, and *atmc9‐1* mutants, we found that losing *At*MC4 expression reduced wound‐induced, differentially expressed genes (DEGs) by over 97%, while *At*MC9 loss decreased DEGs by only 20% (Figure 2). Among DEGs with high significance (Log2 FC >2), 341 depended specifically on *At*MC4, whereas only three required *At*MC9 alone. Additionally, 233 DEGs were co‐dependent on both *At*MC4 and *At*MC9, as they were not induced in either mutant. Interestingly, 118 DEGs were induced only in the *atmc9‐1* background and not in WT or *atmc4‐1* plants, suggesting *At*MC9 represses them in WT plants during wounding and prevents these genes from being activated. Our analysis also identified 75 DEGs significantly repressed by wounding (Log2 FC <−2) in WT plants, with 19 specifically needing *At*MC4 and 56 requiring both MCs (Figure S3).

Gene ontology (GO) analysis of highly induced DEGs (Log2 FC >3) categorized them into MC4‐dependent (231 genes) and MC4/9 co‐dependent (141 genes) groups. MC4‐dependent genes were enriched in pathways related to stress response, abiotic stress, defense, wounding, phytohormones, metabolism, and development, while MC4/9 co‐dependent genes showed similar enrichments but had fewer development‐related genes, which were mostly associated with senescence (Tables S2 and S3). These findings indicate *At*MC4 is the primary driver of transcriptional changes in response to wounding, activating defense as well as developmental pathways. *At*MC9 plays a supportive role, aiding *At*MC4 in activating additional defense and senescence‐related genes while repressing others in WT plants. These findings indicate a coordinated mechanism by these two MCs for balancing stress response, immunity, and growth in wounded tissues.

### Wounding‐induced genes regulated by a Propep1‐ *At*MC4‐Pep1 module

The phytocytokine Pep1 is known to activate basal immunity (Bartels et al., 2013; Ge et al., 2022; Yamada et al., 2016), with *At*MC4 processing Propep1 into the active elicitor Pep1 (Hander et al., 2019). We sought to determine the percentage of genes among the *At*MC4‐dependent DEGs that are dependent on Pep1 maturation via cleavage of Propep1. We reasoned that genes which solely rely on Pep1 signaling through the known PEPR1/2 and BAK1 receptors (Schulze et al., 2010; Yamada et al., 2016) should be epistatic to the loss of *AtMC4* when treated with synthetic Pep1, as this peptide will not require processing by *At*MC4, unlike recombinant Propep1. To this end, we analyzed transcriptomes from WT, *atmc4‐1* and *atmc9‐1* *Arabidopsis* leaf tissues infiltrated with water, 0.1 μM synthetic Pep1, or 0.1 μM recombinant Propep1 followed by compression wounding to activate endogenous MCs.

**Figure 3:**
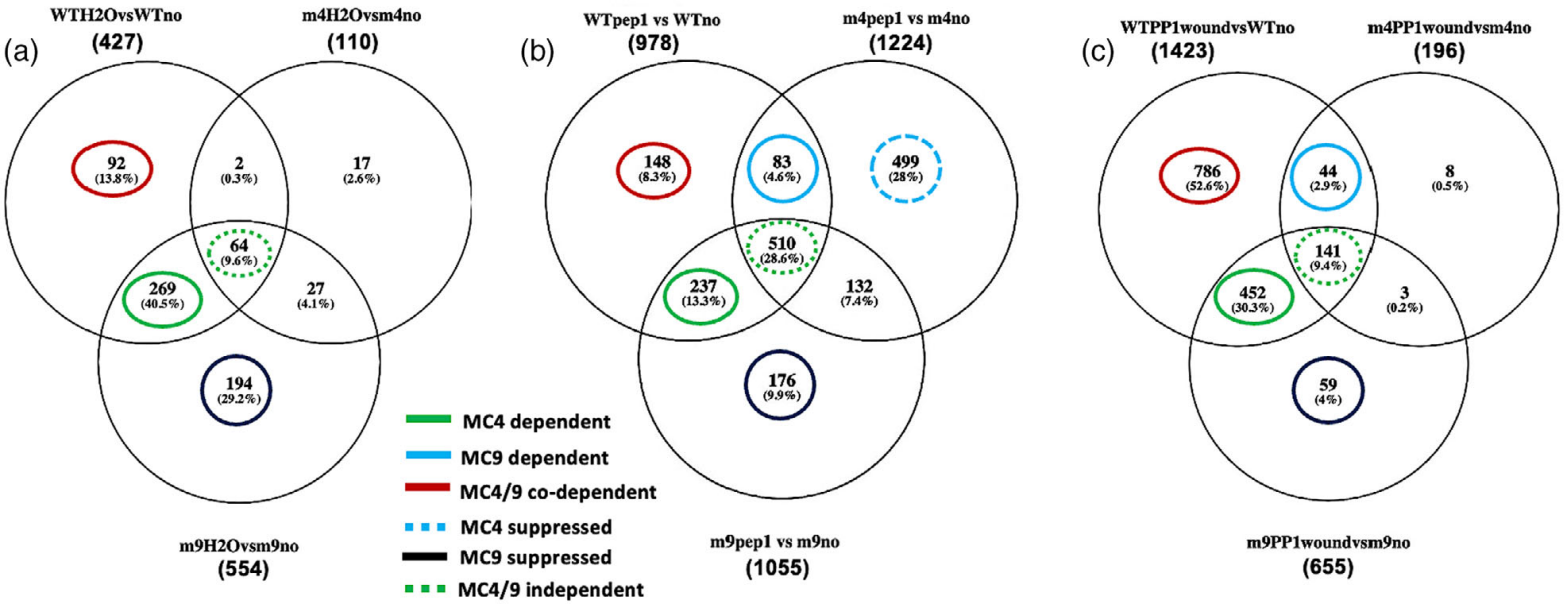
Genes induced by water, Pep1, or Propep1 infiltration in *Arabidopsis*. (a) Wounding via water (H_2_O) infiltration, (b) Pep1 infiltration, and (c) Propep1 (PP1) infiltration with subsequent forceps wounding treatment in WT, *atmc4‐1*, and *atmc9‐1* leaves, respectively. Wounding involved multiple leaf compressions with forceps. PP1 represents infiltrating leaves with 0.1 μM of purified recombinant Propep1 for 2 h before mechanical wounding with forceps for another 2 h; Pep1 represents infiltration of leaves with 0.1 μM synthetic Pep1. After 4 h, samples were collected, snap frozen in liquid nitrogen, and stored at −80°C for RNA preparation. DEGs with Log2 FC >3, *P*_adj_ <0.05 are used for the Venn analysis. Total DEGs in each group are shown in parentheses.

We found water infiltration alone induced a wound‐like transcriptome response, similar to forceps compression, albeit with some differences (Figure 3a; Figure S4). Of the 427 highly significant DEGs, 85% required *At*MC4 (63% specifically and 22% co‐dependent with *At*MC9). Pep1 infiltration increased DEGs to 978 (Log2 FC >3), with 52% (510) independent of MCs. However, some Pep1‐induced DEGs still required *At*MC4, *At*MC9, or both (Figure 3b). Notably, a large group of Pep1‐induced genes (499 DEGs) that are repressed by *At*MC4 in WT plants is revealed in the *atmc4‐1* genotype. Propep1 infiltration with compression wounding tripled the number of DEGs compared with water alone, with 87 and 58% of these DEGs suppressed in *atmc4‐1* and *atmc9‐1*, respectively (Figure 3c), confirming MC‐dependent Propep1 processing.

Compared with forceps wounding, Pep1 or Propep1 plus wounding treatments induced 3–4 times more DEGs (Figure S5), and may indicate that even at 0.1 μM, this treatment could be confounded by its ectopic nature with respect to the concentration and spatial distribution of the cognate phytocytokine Pep1. Nevertheless, the DEG datasets obtained with Pep1 and Propep1 treatments can be used to identify the common gene set between groups that are Pep1 or Propep1 inducible and those that are *At*MC4‐dependent DEGs under compression wounding treatment by forceps alone. Leveraging the data presented in Figure 3 and Figures S6 and S7, we estimated that 128 of the 377 (34%) wound‐induced DEGs (Log2‐FC >3) are *At*MC4‐dependent and mediated by Pep1 production (Figure 2b; Table S4). In contrast, 244 (64%) *At*MC4‐dependent DEGs (Table S6) were Pep1‐independent, suggesting activation mediated via *At*MC4 substrates other than Propep1.

GO analysis of the 128 Pep1‐responsive (Pep+) DEGs identified 45 significant terms (Table S5), while the 244 Pep1‐independent (Pep−) DEGs yielded 85 terms (Table S7). Both groups shared 31 GO terms, predominantly stress response‐ and defense‐related, including “response to wounding” (GO:0009611). The Pep+ group has 14 unique stress response terms, while the Pep− group has 54 unique terms associated specifically with development, phytohormone functions, insect defense regulation, and antimicrobial processes (Table S8). This suggests the Pep1− group not only enhances defense functions but is also involved in tissue repair and regeneration through activation of phytohormone synthesis, transcription factors (TFs), and stem cell reprogramming at the wounded tissue.

### Comparison of wound‐activated DEGs reveals distinct phytocytokine and transcription factor networks

We compared gene categories between Pep1‐responsive (Pep1+) and Pep1‐independent (Pep1−) *At*MC4‐regulated DEGs to understand their roles in wound response. Six categories were selected for our targeted analysis: (1) phytocytokine and secreted peptide‐related functions, (2) transcription factors (TFs), (3) cell wall modification functions, (4) auxin‐related functions, (5) other phytohormones such as JA, GA, and ethylene, and (6) defense‐related.

For the Pep1+ group, 46 of 128 DEGs fit these six categories, with TFs (21) and defense‐related genes (Huot et al., 2014) dominating (Table 1). Few DEGs were linked to growth‐related functions like phytohormones or cell wall modification. Two Pep1+ DEGs that are highly induced over 360‐fold within 4 h are CEP (C‐terminally encoded peptide) 14 and PROSCOOP 7. CEPs are secreted peptides that can affect root development as well as abiotic stress responses (Liu et al., 2021; Roberts et al., 2013), while PROSCOOPs are more recently discovered phytocytokines that can mediate microbial and herbivore‐related defenses (Rhodes et al., 2021). The TFs among this DEG group include 5 WRKYs, 6 ERFs (Ethylene Response Factors), and an assortment of other classes. Both WRKYs and ERFs are large plant‐specific TF families, known for their roles in stress responses and defense (Table S9). Of the 11 defense‐related genes, the top three DEGs are IOS1 (an LRR‐receptor‐like kinase), NHL10 (NDR1/HIN1 like protein) which is known to be highly induced by Pep1 and other immunity‐inducing elicitors, and an endochitinase (At2G43620) that likely provides anti‐fungal activities. These genes showed 8000‐ to 700‐fold induction. In summary, Pep1+ DEGs activated through *At*MC4 appear to primarily drive immunity and stress response functions.

**Table 1:** Comparison of two subgroups of *At*MC4 regulated genes for wounding response. Selected categories of DEGs for Pep1‐responsive (A) and Pep1‐insufficient (B) genes are shown

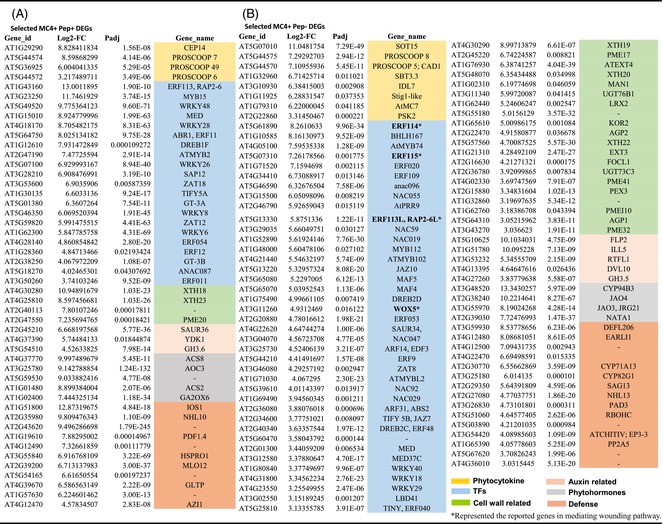

Color key for highlight: Orange: phytocytokine related; blue: transcription factors; green: cell wall modification related; pink: auxin related; gray: phytohormone related; salmon: defense related.

For the Pep1− group, 91 of 244 DEGs fit within the six categories, with more phytocytokine (8 DEGs) and cell wall modification (20 DEGs) related genes, compared with four each in the Pep1+ DEGs (Table 1). Phytocytokine DEGs included PROSCOOP5, PROSCOOP8, peptide phytosulfokine 2 (PSK2), IDL7 (IDA‐like 7), and a Stig1 family peptide. A subtilase (SBT3.3), potentially processing PROSCOOPs (Yang et al., 2023), and *At*MC7 (a type II MC, >70‐fold induced) that could process additional Propeps (Figure 1a), were among this group. IDL7 (IDA‐like 7), a secreted peptide which is known to be rapidly induced by basal immunity activation via peptide elicitors that could help to suppress stress‐related ROS signaling (Vie et al., 2017), and a DEG encoding one member of the Stig1 (Stigma‐specific) family of cysteine‐rich peptides that includes the GRIp peptide are both highly induced genes in this group as well. The 20 cell wall functions‐related DEGs (e.g., encoding extensins and pectinesterases) suggest a role in growth and tissue reprogramming in the wounded tissue.

The 39 TFs also comprise a major category for Pep1‐ DEGs, but its composition in terms of representation by specific TF classes diverged from those in the Pep1+ group: a striking shift is in fewer WRKY TFs, with only 3 DEGs, while a surge in the number for another plant‐specific family of TFs in the NAC class increased to 7 from 1 in the Pep1+ group. NAC TFs are a large family of plant‐specific TFs that are known to regulate growth, differentiation, and abiotic stress responses (Olsen et al., 2005). Similarly, ERF TFs increased to 11, including *ERF109*, *ERF114*, *ERF115*, and *ERF113L*, all implicated in mediating wound signaling via jasmonate (Zhang et al., 2019), and found as top DEGs that are activated in a study of hypocotyl wounding responses (Ikeuchi et al., 2017) as well as being important for callus formation at the cut site (Table S9). Along with the observed DEGs for Wuschel‐related Homeobox 5 (WOX5), a key developmental TF for cell fate determination (Pi et al., 2015; Sugimoto et al., 2010), the similarity of our results to previous work confirms and extends the importance of these DEGs for tissue regeneration in diverse wounding assays. In sum, the changes in the pattern of TFs being mobilized in the Pep1‐ group of *At*MC4‐regulated DEGs are consistent with the orchestration of wound healing and regeneration pathways via activation of genes among this group of DEGs during the wound response.

### Type II metacaspases shape Pep1‐driven immune transcriptome

The bacterial flagellum‐derived flg22 peptide, a microbe‐associated molecular pattern (MAMP), activates basal immunity in Arabidopsis via the FLS2 receptor, while Pep1 uses PEPR1/2 receptors. Both of these ligand/receptor pairs share the BAK1 adaptor receptor kinase, which may explain their overlapping transcriptomic responses (Bjornson et al., 2021; Schulze et al., 2010; Yamada et al., 2016). Despite their similarities, the differences between the two transcriptome profiles induced by these peptides of distinct origin could be important for affecting differential outcomes, that is, a strong bacterial pathogen immunity induction in response to flg22, and a more tempered defense activation upon Pep1‐induced wound response to minimize antagonism on growth and developmental functions. To examine if *At*MC4 and *At*MC9 modulate Pep1's defense transcriptome, we compared Pep1‐ and flg22‐induced DEGs in WT, *atmc4‐1*, and *atmc9‐1* genetic backgrounds (Figure S8).

In WT, both elicitor peptides induced about 850 DEGs compared with water, with 538 shared and about 330 unique to each. In *atmc4‐1*, Pep1‐induced DEGs increased by almost 400, likely due to loss of *At*MC4‐mediated suppression, while flg22‐induced DEGs showed little change. In contrast, for *atmc9‐1* plants, flg22‐induced DEGs rose by about 400 genes, adding 205 shared and 177 flg22‐specific DEGs, while Pep1‐specific DEGs dropped by over 50%, suggesting *At*MC9 is necessary for their induction (Figure S8c). This indicates *At*MC9 significantly represses some flg22‐responsive immunity genes (possibly to minimize growth inhibition) by modulating the strength of the defense response (Huot et al., 2014; Major & Campos, 2022).

To examine if *At*MC4 and *At*MC9 tailor the defense‐related transcriptomic landscape activated by Pep1, we analyzed de‐repressed DEGs in *atmc4‐1* (721 genes) and *atmc9‐1* (194 genes) following Pep1 treatment. We focused on four categories: phytocytokine‐related genes that encode secreted peptides and related functions, ERF TFs, WRKY TFs, and non‐coding RNAs (ncRNAs) that are prominent among the elicitor‐induced DEGs (Table 2). These categories may represent key defense functions and could be involved in signal amplification via transcriptional controls. De‐repressed phytocytokine‐encoding DEGs (*RGF7*, *PSK1*, and *CLE12*) in either mutant were flg22‐specific in WT, suggesting *At*MC4 and *At*MC9 could suppress flg22‐responsive genes to fine‐tune Pep1's immune response. Similarly, an additional five *At*MC4‐suppressed and four *At*MC9‐suppressed DEGs were flg22‐specific in WT (more details can be found in Supplemental Information). These observations are consistent with the interpretation that *At*MC4 and *At*MC9 can help shape Pep1's immune transcriptome by modulating defense‐related genes downstream of the PEPR1/2‐BAK1 receptor kinase complex.

**Table 2:** Comparison of select gene categories between shared and elicitor‐specific DEGs (A), and de‐repression of Pep1 activatable genes in two *At*MC mutant backgrounds (B)

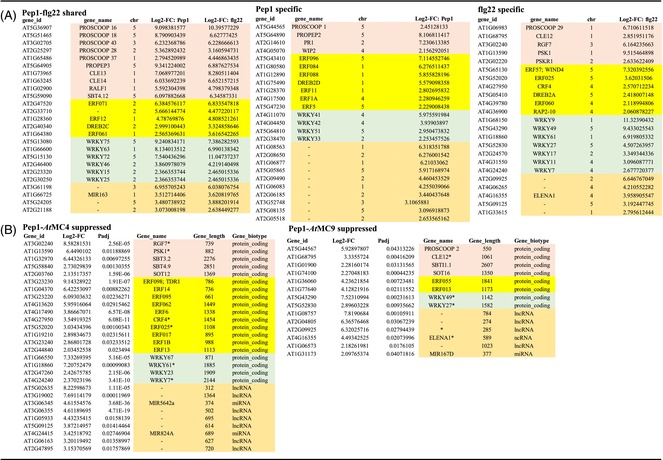

Color key for highlight: salmon: phytocytokine‐related; yellow: ERF TFs; green: WRKY TFs; orange: non‐coding RNAs. In panels of B, DEGs with asterisks denote DEGs that are induced by flg22 in the WT background.

### Validation of *At*MC4‐dependent and wound‐induced genes in *atmc4‐1* mutants

Our transcriptomic analysis identified four distinct groups of wound‐induced, *At*MC4‐dependent DEGs. Group 1 DEGs (e.g., *ACS8* and *WRKY48*) rely solely on *At*MC4, while Group 2 (e.g., *PDF1.4* and *NHL10*) requires both *At*MC4 and *At*MC9. These four genes all have known functions in stress and defense responses, and each is induced by more than 50‐fold under our wounding treatment using RT‐qPCR assays to validate the RNA‐seq results (Figure S9). In contrast to Groups 1 and 2 genes, Groups 3 and 4 genes are wound‐induced DEGs that are not responsive to Pep1 treatment and thus may represent targets of cryptic peptide signals whose pro‐peptide precursors are processed by *At*MC4 specifically (Group 3) or by *At*MC9 as well (Group 4). Examples of genes in these two categories are *WOX5* and *AtMC7*, respectively (Figure S9).

**Figure 4:**
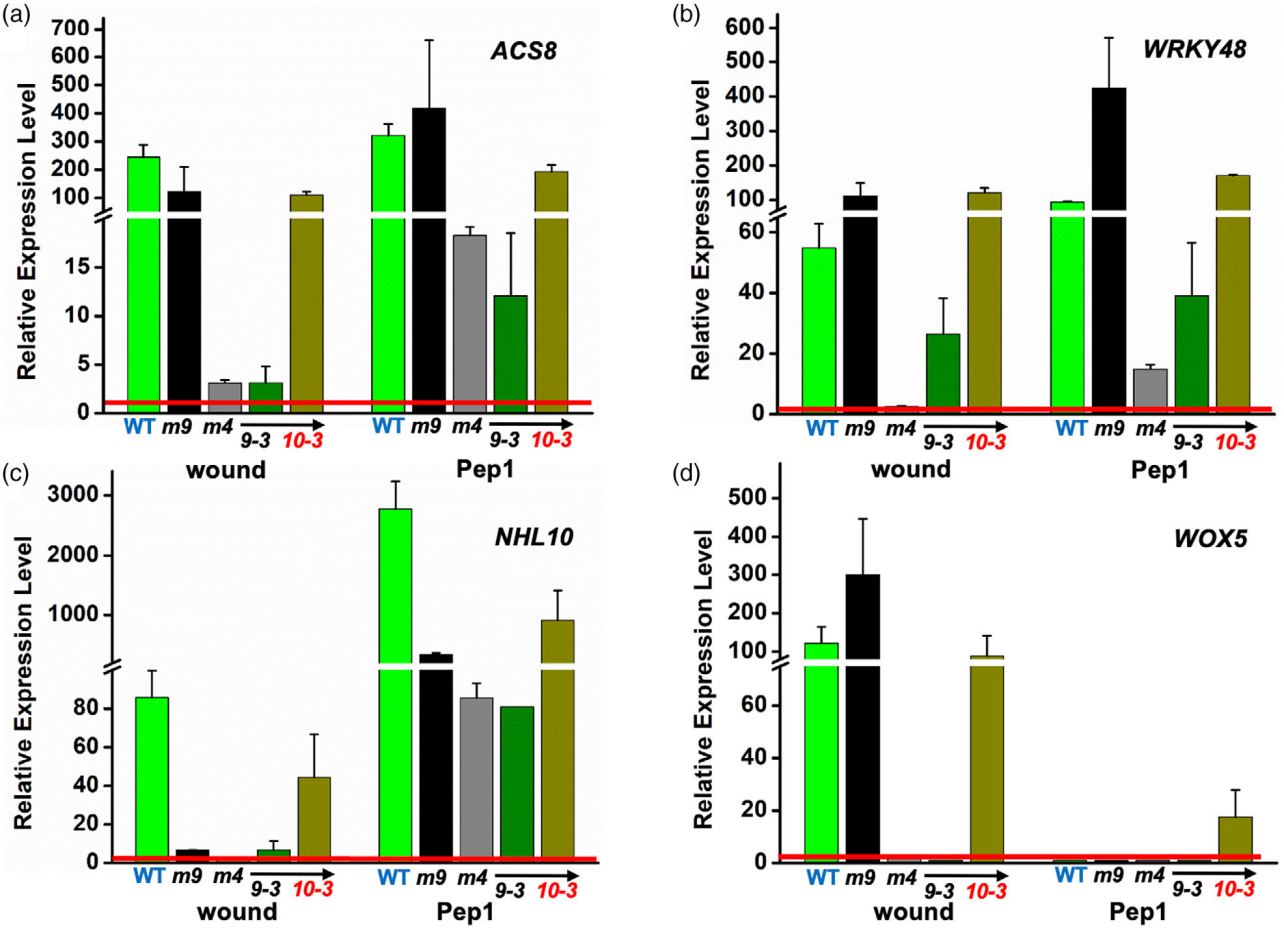
Marker gene responses to wounding and Pep1 in different genetic backgrounds. RT‐qPCR analysis of steady‐state transcript levels for four marker genes that respond to wounding is compared in different genetic backgrounds: WT (Green), *atmc9‐1* (black), *atmc4‐1* (dark gray), transgenic plants *MC4pro::MC4 in atmc4‐1* T3 line #*9‐3* (olive green), and line #*10‐3* (dark yellow). Expression level for each gene (a) *ACS8*, (b) *WRKY48*, (c) *NHL10*, and (d) *WOX5* was shown as fold change relative to no treatment control (red line). The *UBC21/PEX4* (At5G25760) gene was used as the reference. Bars show the standard deviation from triplicate samples. Different scales optimize the data resolution for each gene. Red bars show the value of 1 for no change. Wounding with forceps (wound) and treatment with Pep1 synthetic peptide at 0.1 μM (Pep1).

To test and confirm *AtMC4's* roles, we generated transgenic *atmc4‐1* lines expressing the *A*tMC4 coding sequence under a 1.5 kb *AtMC4* promoter (Figure S10). Two independent transgenic lines (Figure S10), #9‐3 (low expression) and #10‐3 (high expression), were selected for wound‐induced gene expression studies with a set of selected marker genes from the different transcript response groups defined above. Line #10‐3 fully restored wound induction of *ACS8*, *WRKY48*, *NHL10*, and *WOX5*, while line #9‐3 partially restored *WRKY48* expression, but not *ACS8*, *NHL10*, or *WOX5*. *ACS8* and *NHL10* can be induced by Pep1 treatment in the *atmc4‐1* and *atmc9‐1* backgrounds, albeit at diminished levels in *atmc4‐1* than in WT; with the transgenic line #10‐3 more closely matched to WT levels (Figure 4). For *WRKY48*, Pep1 induction was 20‐fold lower in *atmc4‐1* but restored in line #10‐3, confirming *AtMC4's* role downstream of Pep1. Interestingly, Pep1 induction of *WRKY48* increased by about fourfold in the *atmc9‐1* mutant background, suggesting that the expression of this TF encoding gene may be targeted by *At*MC9 for suppression during Pep1‐mediated defense signaling. As expected, *WOX5* showed no significant Pep1 induction (Figure 4).

**Figure 5:**
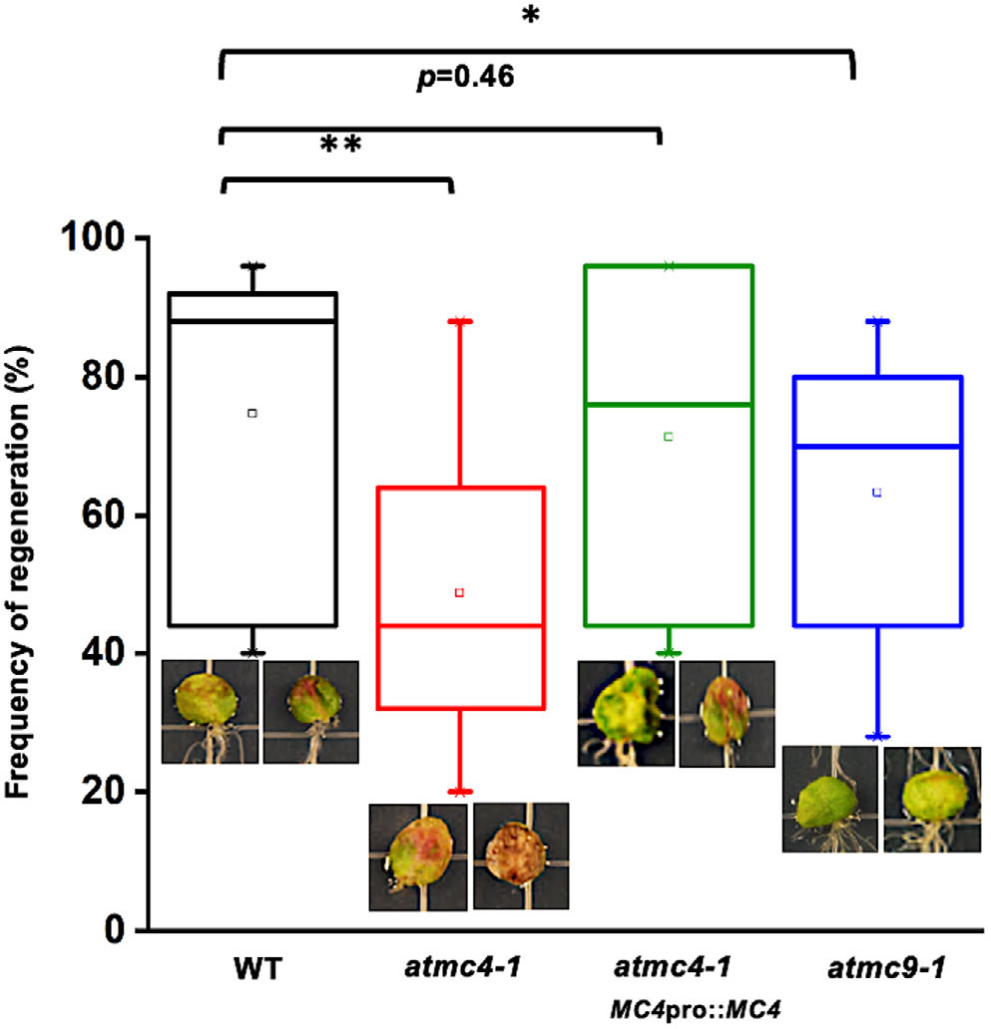
*AtMC4* is required for root regeneration from excised leaves in *Arabidopsis*. Root regeneration frequency assay in WT, *atmc4‐1*, *atmc9‐1*, and transgenic *AtMC4*pro::*AtMC4* in *atmc4‐1 #*10‐3. Statistical analysis of root regeneration frequency in the four genetic backgrounds. *P < 0.05, **P < 0.01.

In view of the clear requirement for *At*MC4, and to a lesser extent *At*MC9, for orchestrating the wound response transcriptome in leaf tissues, we predicted that there should be a significant impact on tissue regeneration in damaged leaves of *atmc4‐1* plants. Using the root‐from‐leaf assay described previously (Hernandez‐Coronado et al., 2022), we compared the frequency of root regeneration from the wound site in excised Arabidopsis leaves taken from plants with different genetic backgrounds. We found that leaves from the *atmc4‐1* mutant displayed a significant decrease in the frequency of root regeneration to about half of that observed for WT plants (Figure 5). Moreover, *atmc4‐1* plants displayed much higher levels of red pigments—most likely flavonoids, which could indicate a stronger response to the wounding stress (Figure 5; Figure S11). In contrast, leaves from the *atmc9‐1* background showed a decrease of only about 20% in root regeneration compared with WT along with lower statistical significance (Figure 5), and without excess flavonoids being observed (Figure 5; Figure S11). Importantly, transgenic line #10‐3 in the *atmc4‐1* background complemented these mutant phenotypes (Figure 5), thus confirming the importance of *At*MC4 for root regeneration from the wounded petiole.

### Ectopic *At*MC9 expression partially rescues *atmc4‐1* wounding response defects in transcript changes

The successful complementation of the *atmc4‐1* mutant using a synthetic *AtMC4* transgene enabled us to use this expression cassette to determine if there is functional redundancy between *At*MC4 and *At*MC9, since both MCs can process Propep1 *in vitro* (Liu et al., 2025). We generated a gene expression construct for *At*MC9 using the same expression vector as used in the *atmc4* complementation experiments above. This construct was transformed into the *atmc4‐1* mutant background. One highly expressing transgenic line, *AtMC4*pro::*AtMC9*, #10 (Figure S12), was selected from over 12 independent transformants for further studies to assess the ability of *At*MC9 to rescue wound‐ and Pep1‐induced gene expression. Although *At*MC4 and *At*MC9 could process Propep1 *in vitro*, they differ in their enzymatic activation mechanisms, with *At*MC4 having a strict requirement for calcium (Fortin & Lam, 2018; Hander et al., 2019; Watanabe & Lam, 2011b) and *At*MC9 being activated only by acidic pH conditions and independent of calcium (Fortin & Lam, 2018; Vercammen et al., 2004). In addition, these two MCs can also have other distinct target substrates, as demonstrated above in the case of Propep3.

**Figure 6:**
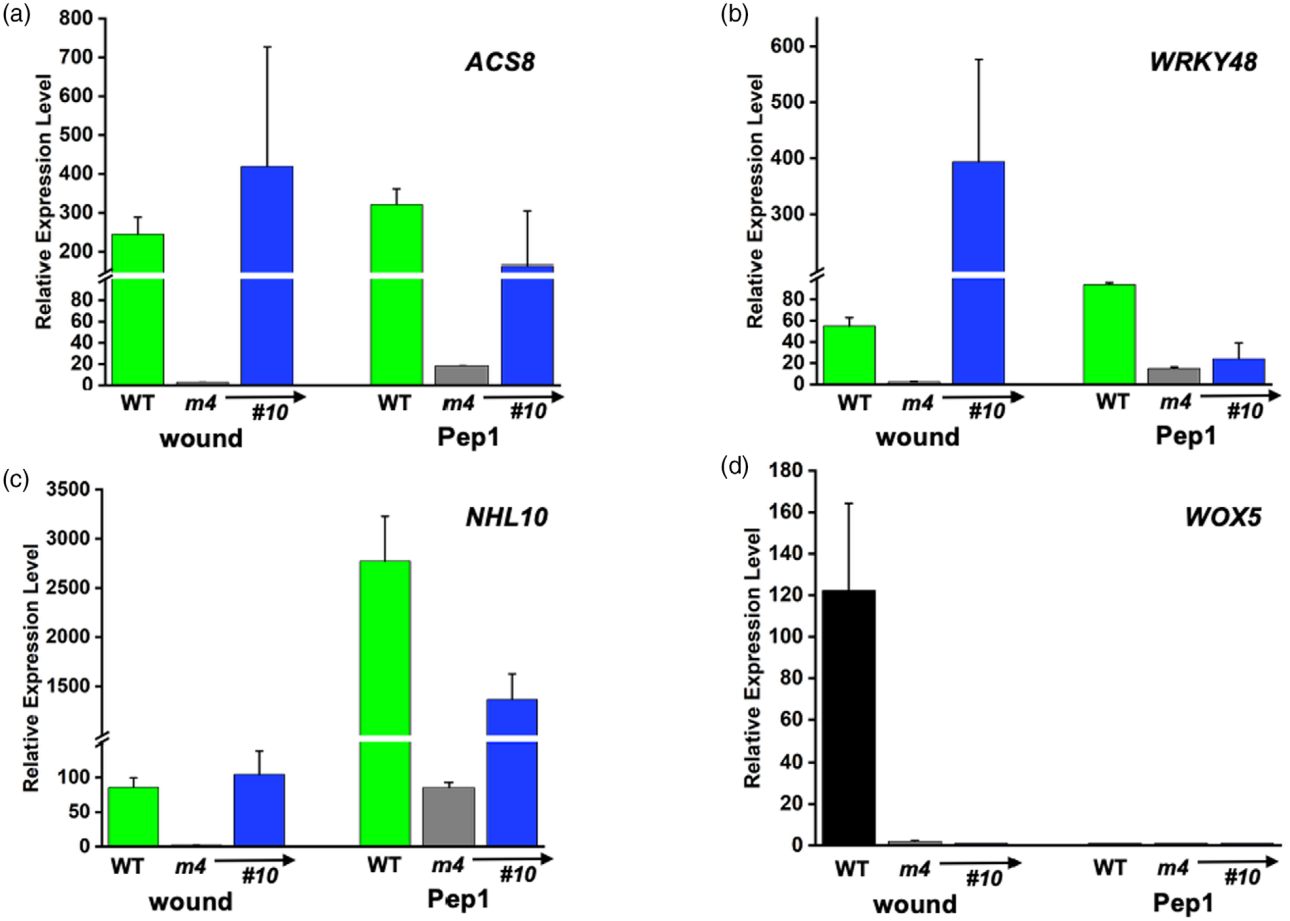
Ectopic *At*MC9 expression partially rescues *At*MC4‐dependent wound response. Leaves from WT, *atmc4‐1* (*m4*), and transgenic plants *MC4pro*::*MC9 in atmc4‐1*, T3 line #*10* (*#10*) were either wounded by forceps or infiltrated with synthetic Pep1. Total RNA from the treated samples was assayed by RT‐qPCR for specific genes of interest. The *UBC21/PEX4* (At5G25760) gene was used as the normalizing reference. The change in expression level for each gene (a) *ACS8*, (b) *WRKY48*, (c) *NHL10*, and (d) *WOX5* was calculated from the ratio to no treatment control, respectively. Bars show the standard deviation from triplicate reactions. Different scales optimize the data resolution for each gene. Wounding with forceps (wound) and treatment with Pep1 at 0.1 μM (Pep1) are as described in “Materials and Methods” section.

Ectopic *At*MC9 expression recovered wound‐induced expression of Pep1‐responsive (Pep+) genes, such as *ACS8*, *WRKY48*, and *NHL10* in *atmc4‐1* background, but did not restore expression of the Pep1‐independent (Pep−) gene *WOX5* (Figure 6). Interestingly, although Pep1‐induced gene expression was also rescued for *ACS8* and *NHL10*, the activation of *WRKY48* was not. This striking distinction correlates with the observation that *At*MC9, but not *At*MC4, can repress *WRKY48* expression downstream from the Pep1‐mediated signaling cascade as noted above (Figure 4). Thus, ectopic *At*MC9 expression likely compensates for *AtMC4's* Propep1 processing but at the same time increases *WRKY48* suppression. The inability of ectopic *At*MC9 expression to rescue wound induction of *WOX5* suggests *AtMC4's* unique role in activating Pep1‐independent, wound‐responsive genes and implicates cryptic *At*MC4‐specific substrates that are needed for wound induction of this key developmental regulator.

## DISCUSSION

### Type II metacaspases orchestrate wound‐induced transcriptome for immunity and regeneration

Using a transcriptomic approach, this study provides a comprehensive analysis of transcription‐mediated pathways that could be impacted by mechanical wounding of leaf tissues beyond the Pep1‐mediated defense signaling module. Comparative analysis with *atmc4‐1* and *atmc9‐1* mutants reveals *At*MC4 as a key regulator that orchestrates transcript changes for stress response, immunity as well as developmental pathways. In contrast, *At*MC9 acts to suppress a subset of basal immunity genes that are normally activated by MAMPs such as flg22, thus helping to modulate the output downstream of the PEPR1/2‐BAK1 receptor complex upon its activation by Pep1. Cooperation between *At*MC4 and *At*MC9 is essential for inducing defense and abiotic stress‐related Group 2 genes, alongside the *At*MC4‐dependent Group 1 genes. This co‐dependence may derive from the existence of common proteolytic substrates, like Propep1 (Figure 1a; Liu et al., 2025; Shen et al., 2019), despite their distinct mechanisms of activation, with *At*MC4 requiring local calcium levels in the millimolar range while *At*MC9 requiring a pH of 5.6 or lower to initiate autolytic cleavage and associated conversion from zymogens into active proteases.

**Figure 7:**
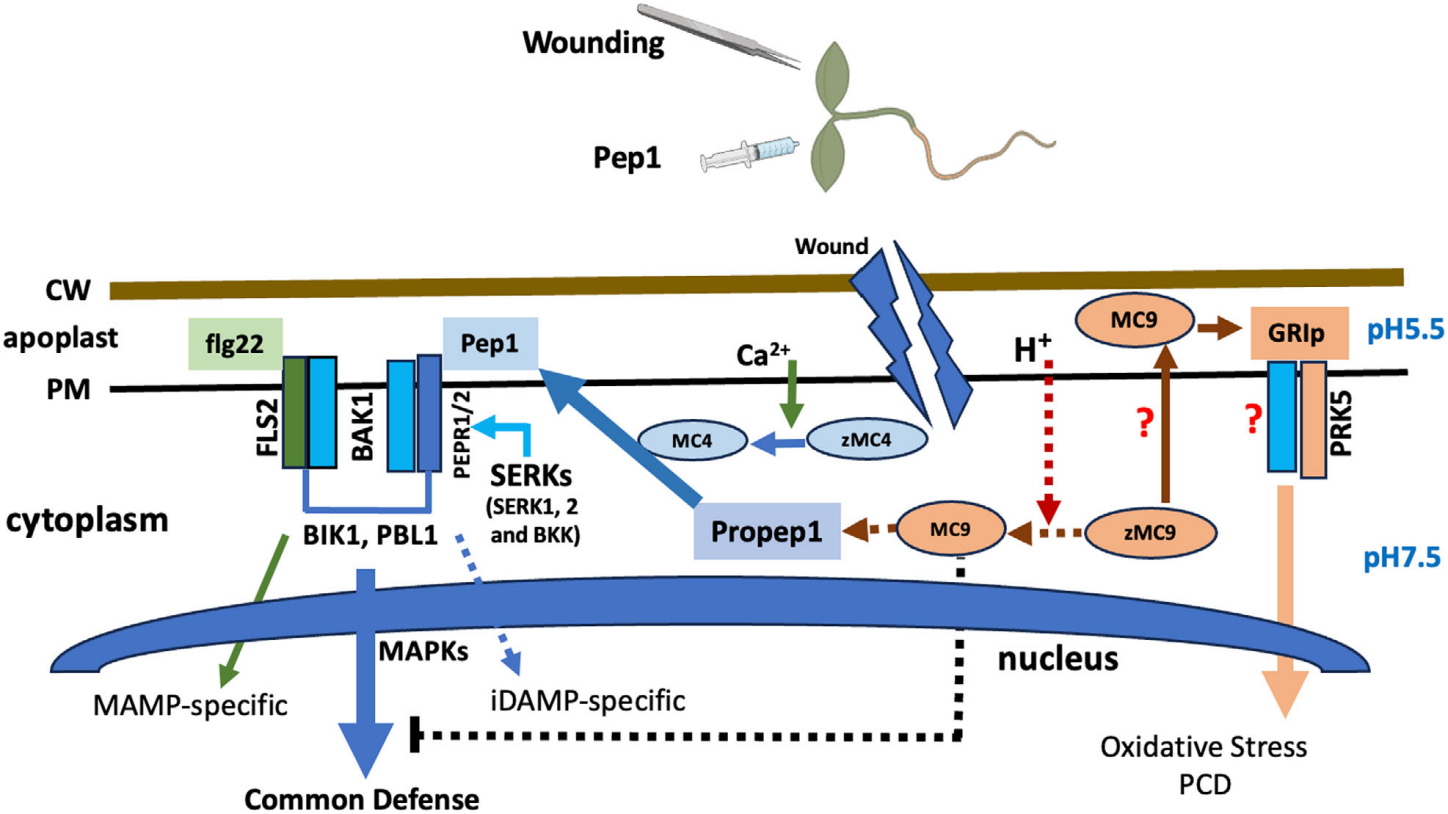
An updated model for type II metacaspase functions in damage‐associated molecular pattern (DAMP) signaling. Current known components for *At*MC4 (MC4) and *At*MC9 (MC9)‐mediated phytocytokine conversion and signaling components in basal immunity activation. BIK1: Botrytis‐induced kinase 1; PBL1: PBS1 (AvrPPHB Susceptible‐1)‐Like 1 RLCK. The role of BAK1/SERK3 and BKK/SERK4 as adaptor kinases to mediate PRK5 function has been speculated but not proven. In addition, how zMC9 or MC9 may be translocated into the apoplastic space to generate GRIp is unknown as well. Solid lines indicate previously known functions while broken lines denote new activities/information revealed in this study.

Figure 7 presents a model that integrates our present findings with prior knowledge on Pep1 signaling and metacaspase properties. Previous work by Hander et al. (2019) showed that cytosolic Propep1, which is anchored to the surface of the vacuole, is targeted for cleavage by *At*MC4 after activation of its zymogen (zMC4) in the cytosol through the calcium influx that occurs upon wounding to release Pep1. How the zymogen of *At*MC9 (zMC9) may be activated during wounding remains unclear. While *At*MC9 has been shown to be required to generate the PCD‐inducing peptide GRIp, which is found in the apoplast (Wrzaczek et al., 2015), the sub‐cellular location where this conversion occurs remains unknown. Similarly, ectopically expressed *At*MC9 fusion proteins have been reported to be present in the cytoplasm, nucleus, and apoplast under ambient conditions (Tsiatsiani et al., 2013; Vercammen et al., 2006). Our finding that *At*MC9 expressed under control of the *AtMC4*pro could partially rescue *atmc4‐1* defects and recover wound induction of many Pep1‐dependent genes indicates that cleavage of intracellular Propep1 can be performed by *At*MC9 in place of *At*MC4. This novel finding supports the possibility that zMC9 could be activated by transient and localized proton spikes within the cytosol during wounding or following the induction of basal immunity by peptide elicitors (Bosch & Franklin‐Tong, 2024; Li et al., 2021), which could then enable it to cleave cytosolic Propep1 (Figure 7).

### *At*MC4 mediates bifurcating pathways to activate defense‐ and tissue regeneration‐related functions upon wounding

A striking finding from this study is the critical importance of *At*MC4 for the global response of wound‐induced genes. In addition to its previously defined Propep1 convertase activity (Hander et al., 2019), which impacts about a third of all high significance, wound‐induced DEGs, *At*MC4 also serves to activate other defense‐related and abiotic stress response pathways that are Pep1‐independent, notably those involved in heat stress as well as oxidative‐ and hypoxia‐stress‐related functions (Tables S3, S5, and S8). Strikingly, it is also a key player in activating an ensemble of genes that are involved in cell growth and differentiation, a result that is consistent with their importance in regeneration during the wound healing process (Table 1; Tables S2, S3, S7, and S9). This is corroborated by the observed decrease in root regeneration from the petiole wound site of excised leaves derived from the *atmc4‐1* mutant and the restoration of root regeneration following complementation of the mutant with the *At*MC4 expression cassette (Figure 5). It is significant that these development‐ and growth‐related genes are enriched in the group that is not mediated by Pep1 production, which implicates cryptic proteolysis targets of *At*MC4 as the likely mediators that activate these regeneration‐related genes (Figure S13). Failure of ectopic expression of *At*MC9 to complement *atmc4‐1* defects in *WOX5* induction indicates these targets would likely be selective between the two MCs and is consistent with the low impact of the *atmc9‐1* mutation on root regeneration from the wound site (Figure 5). The elucidation of these new targets for *At*MC4 should further expand our understanding of the transcriptional signaling network that leads to a successful recovery of wounded plant tissues. Similarly, the finding that *At*MC4 may be additionally needed beyond its Propep1 convertase function to regulate other DEGs that are induced by exogenous Pep1 treatment raises the possible existence of additional cellular targets for *At*MC4 that could assist in the signal amplification beyond activation of the PEPR1/2‐BAK1 receptor complex through Pep binding (Figure S13). Thus, future discovery of novel cellular targets for *At*MC4 should provide candidates to examine these phenomena in more detail.

### Repressive function by *At*MC9 for Pep1‐mediated gene activation helps to shape the defense‐related transcriptome landscape in the DAMP response

While many stress‐related signaling pathways are known to share common adaptor receptor kinases as well as affecting overlapping sets of target genes (Bjornson et al., 2021; Rhodes et al., 2021; Schulze et al., 2010; Yamada et al., 2016), how these pathways can be modulated to produce distinct transcriptional responses and phenotypic outcomes is largely unknown. Our results show that *At*MC9 plays a prominent function to modulate Pep1‐mediated gene activation patterns upon wounding by suppressing a set of genes that are normally expressed strongly during basal immunity induction via the MAMP flg22, which shares with Pep1 in the use of the BAK1 adaptor kinase along with its own specific receptor FLS2 (Yamada et al., 2016). Since these *At*MC9‐suppressed genes are not sensitive to the *atmc4‐1* defect, their suppression by *At*MC9 likely would involve cleavage of specific target substrates for this MC, as shown here for Propep3 which we describe in this work (Figure 1c). It is pertinent to note that Propep3 has previously been shown to be secreted into the extracellular space (Yamada et al., 2016) and thus should be found in the apoplast, where the pH is significantly more acidic than the cytosol and where *At*MC9 may be present and active (Vercammen et al., 2006). Further expansion of our knowledge on the *in vivo* targets of these metacaspases will thus be important to uncover mechanisms through which global transcriptome outputs from similar receptor kinase complexes with common adaptor partners can be modulated to achieve optimal effects under a particular context.

## MATERIALS AND METHODS

### Structure‐based approach to the identification of potential substrate targets for type II metacaspases

To apply a protein sequence‐based approach for rapid curation of potential substrate candidates that may be cleaved by type II MCs, we used the structure prediction algorithm AlphaFold2 (Jumper et al., 2021) to systematically screen the protein sequences from each of the eight *At*Propep for interaction with each of the six type II MC proteins in Arabidopsis. We applied three filters to identify potential targets for each of the type II MCs: (1) using clash score calculated by PHENIX to remove candidates that have more than 50 clashes with the respective MC, (2) the interface area with the particular MC was calculated by the CCP4 program PISA (https://www.ccp4.ac.uk/) using a cutoff of 500 Å^2^ to remove loosely bound proteins, (3) setting the distance between the catalytic residue C139 thiol group (SG) in *At*MC4, or the equivalent active site residue in the other MCs, and the closest protein atom from the candidate Propep target should be less than 4.5 Å away. A further refinement in our analysis pipeline prioritizes a shorter distance between the active site cysteine SG and arginine/lysine carbonyl oxygen of the target substrates as optimal. Lastly, to ensure that the catalytic site of *At*MC4 and the other five type II MCs will not be blocked by their respective linker, this part of the protein sequence was deleted before their structure prediction and docking of the Propep substrates using the pipeline as described. Additional details can be found in the Supplemental Information file.

### Plant materials and growth conditions

*A. thaliana* accession Columbia (Col‐0), its homozygous mutants *atmc4‐1* (SAIL_856_D05), *atmc9‐1* (SALK_075814), transgenic plant lines *AtMC4*pro::*AtMC4* in *atmc4‐1* #9‐3, *AtMC4*pro::*AtMC4* in *atmc4‐1* #10‐3, and the transgenic plant line *AtMC4*pro::*AtMC9* in *atmc4‐1 #*10 were used in this study. The mutant lines *atmc4‐1* and *atmc9‐1* were originally obtained from the ABRC.

The *AtMC4*pro::*AtMC4* vector was constructed by inserting the *At*MC4 coding region fragment into the BamHI and SacI double‐digested lab vector pNW203 (Watanabe & Lam, 2011a). Then the constructed vector was mobilized into the *Agrobacterium* strain GV3101 and used for *Arabidopsis* transformation by flower dipping of *atmc4‐1* plants. Homozygous transgenic lines were identified at the T2 stage. RT‐PCR was performed to verify *AtMC4* transgene expression at the RNA level, and the results were used to select transgenic lines for the phenotyping studies (Figure S9). For construction of the *AtMC4*pro::*AtMC9* vector, a set of adapters (Forward: 5′GATCC CTCGAG GTCGAC GCTCAGC GTTTAAACGAGCT3′, Reverse: 5′CGTTTAAAC GCTGAGC GTCGAC CTCGAGG 3′) was used for annealing with the BamHI and SacI double‐digested lab vector pNW203. The resulting vector was used to insert an *At*MC9 coding sequence fragment. The *AtMC4*pro::*AtMC9* vector was then transformed into *Agrobacterium* strain GV3101 and used for *Arabidopsis* transformation by floral dipping of *atmc4‐1* plants. Homozygous transgenic lines were obtained by the T2 stage. *At*MC9 transgene expression was confirmed by RT‐PCR using a transgene‐specific primer pair before the selection of the transgenic line for phenotyping studies (Figure S11).

For plant growth, seeds were sterilized with 25% regular bleach (Clorox), germinated on Murashige and Skoog salts or ½ X Murashige and Skoog w/o sucrose plates, and then stratified at 4°C for 1–3 days. Plates were then moved to a growth chamber (22°C, 8 h light/16 h dark cycle). For adult plants, 1‐week‐old seedlings were transferred to soil and grown for three additional weeks before use.

### Overexpression, purification and assays with r*At*MC4, r*At*MC9, rGST‐Propep1, rPropep1 w/o tag, and rPropep3‐GST

p*ET23a*‐*At*MC4 and p*ET23a*‐*At*MC9 were transformed into Rosetta™ 2(DE3) *pLysS* cells for the production and purification of the corresponding recombinant proteins, as described previously (Fortin & Lam, 2018). pGEX‐4T‐1‐GST‐TEV‐Propep1 (PP1) vector (Zhu et al., 2020) was used to transform *Escherichia coli* BL21 (DE3) *pLysS* strain for protein production. The sequence of Propep3‐GST was synthesized and cloned into p*ET23a* (+) by the company Genscript (Piscataway, NJ, USA). Overexpression of GST‐TEV‐Propep1 and Propep3‐GST proteins, preparation of the tag‐free rPropep1 protein, and assay with recombinant *At*MC proteins were performed essentially following the protocol previously described (Zhu et al., 2020) with the addition of western blotting to detect the GST fusion peptides specifically using GST‐tag polyclonal antibody (Invitrogen, # A‐5800). More details can be found in the Supplemental Information file.

### Leaves treated with wounding or infiltration with synthetic peptides *in planta*

Leaves of 4‐week‐old Arabidopsis plants from different genetic backgrounds of WT, *atmc4‐1*, and *atmc9‐1* were used to perform wounding, water infiltration, or peptide treatments, respectively. For rPropep1 + wounding treatments, three plants were used for one treatment. Two leaves from each plant were infiltrated with 100 nM rPropep1. After 2 h, the infiltrated leaves were wounded by forceps. After another 2 h, the treated leaves were collected and flash‐frozen in liquid nitrogen immediately for storage at −80°C until use. For compression wounding, water infiltration, or synthetic peptides (Pep1 or flg22) treatments, the leaves at the same age were wounded by squeezing five times with serrated forceps, or infiltrated using a syringe with water only, or 100 nM Pep1 (Synthesized by Biomatik, Purity: 85.49%) or 100 nM flg22 (Synthesized by Genscript, Purity: 97.1%) dissolved in water, respectively. After 4 h, samples were collected and frozen as described above. The experiment was repeated twice with similar results.

### Total RNA extraction and transcriptome analysis

Total RNA was extracted from the collected leaf samples using the mirVana™ RNA Isolation kit (Invitrogen), following the manufacturer's guidelines. Quality‐checked RNA samples were sequenced and initially analyzed by Novogene Ltd., followed by cDNA library construction and sequencing on an Illumina platform. Differentially expressed gene analysis between treatments and no treatment was performed by Novogene Ltd. by using DESeq2 (Love et al., 2014). *P* values were adjusted using the Benjamini and Hochberg method. Absolute log2‐fold change of 2 and a false discovery rate (FDR) of 0.05 were initially set as the thresholds for significant and high‐confidence differential expression. Venn Diagram analysis was performed by using Venny 2.1 (Oliveros, 2007‐2015). Cluster analysis was performed by using gene ontology (GO) Term Enrichment analysis and was carried out by using the tool: https://www.arabidopsis.org/tools/go_term_enrichment.jsp. The test type was chosen as Fisher's exact test and the correction was chosen as calculate false discovery rate (FDR). Additional details on the transcriptome and bioinformatics analyses can be found in the Supplemental Information file.

### RT (reverse transcriptase)‐quantitative PCR analysis and root‐from‐leaf regeneration assay

Total RNA was isolated with the above method. TURBO DNA‐free™ kit was used for the removal of DNA from the RNA sample. High‐capacity cDNA reverse transcription kit (Applied Biosystems™) was used to generate cDNA. The generated cDNA was used as the template, and the primers designed for the marker genes were listed in Table S1. Applied Biosystems™ PowerSYBR Green PCR Master Mix and StepOne Plus Real‐Time PCR Station (Applied Biosystems) were used to carry out RT‐qPCR analysis following the manufacturer's protocol for quantifying the relative transcript levels from the gene of interest. For a control reference gene, we use the transcript for At5G25760 encoding UBC21 (ubiquitin‐conjugating enzyme 21)/PEROXIN4 as previously recommended for transcript normalization (Czechowski et al., 2005).

For the root‐from‐leaf regeneration and statistics analyses (Hernandez‐Coronado et al., 2022), seeds of *A. thaliana atmc4‐1*, *atmc9‐1*, their WT accession Col‐0, and transgenic plant lines *AtMC4*pro::*AtMC4* in *atmc4‐1* #10 were plated on ½ X MS medium, with 0.5% sucrose, and stratified for 2 days at 4°C. Then the plates were moved to the growth chamber (22°C, 16 h light/8 h dark cycle) for 15 days. Detached leaf explants were transferred to ½ X MS plates and grown for 15 days under the above conditions. Photos were taken and the number of leaves with root regeneration was recorded for each plate. The Chi‐squared test and Fisher's combined probability test were used to compare the significant differences among samples from different genetic backgrounds.

## AUTHOR CONTRIBUTIONS

Conceptualization: EL, QL. Methodology: ZP, HL, QL. Investigation: ZP, HL. Visualization: ZP, EL, HL, QL. Funding acquisition: EL, QL. Project administration: EL, QL. Supervision: EL, QL. Writing—original draft: EL. Writing—review and editing: EL, QL, ZP, HL.

## CONFLICT OF INTEREST

Authors declare that they have no competing interests.

## Supporting information


**Notes S1.** Pertinent information related to type II metacaspases in plants.
**Methods S1.** Additional Materials and Method Details.

**Table S1.** List of primers used in this study.
**Table S2.** Overrepresented gene ontology (GO) terms for *At*MC4‐dependent upregulated genes in wounding leaf tissues compared with no treatment.
**Table S3.** Overrepresented gene ontology (GO) terms for *At*MC4 and *At*MC9 co‐dependent upregulated genes in wounding leaf tissues compared with no treatment.
**Table S4.** Curated DEGs which are *At*MC4‐dependent and Pep responsive (Pep+).
**Table S5.** GO analysis of Pep+ DEGs.
**Table S6.** Curated DEGs which are *At*MC4‐dependent but not Pep1‐inducible (Pep−).
**Table S7.** GO analysis for Pep− DEGs.
**Table S8.** Combined Pep+ and Pep− GOs sorted for common and specific groups.
**Table S9.** Annotated functions for transcription factor (TF) encoding DEGs from the Pep+ and Pep− groups.

**Figure S1.** Supporting data for confirmation of the predicted cleavage and target sequence of Propep3 by *At*MC9, but not *At*MC4.
**Figure S2.** Workflow and experimental design for identification of metacaspase‐dependent genes via wounding and Pep1 signaling.
**Figure S3.** *At*MC4 is also a key mediator for transcriptional repression upon wounding in leaves.
**Figure S4.** Heatmap analysis shows similar transcriptional response to two physical treatments in *Arabidopsis thaliana* leaf tissues.
**Figure S5.** Summary of genes induced (log2‐fold change >2, *P*_adj_ <0.05) by various treatments in three genetic backgrounds.
**Figure S6.** Overlap of Pep1‐induced DEGs with MC4‐dependent genes upon infiltration reveal gene set that is activated via a Propep1‐*At*MC4‐Pep1 signaling module.
**Figure S7.** Initial curation of wounding‐induced DEGs that are modulated by *At*MC4.
**Figure S8.** Overlap of transcriptional response between infiltration wounding, flg22, and Pep1 treatments in three different genetic backgrounds.
**Figure S9.** Validation of selected reporter marker genes for four distinct DEG groups using RT‐qPCR.
**Figure S10.** Complementation of *atmc4‐1* via transgenic expression of *AtMC4* cDNA under its cognate promoter.
**Figure S11.** Phenotypes of root‐from‐leaf assay in different genetic background.
**Figure S12.** Verification of the *At*MC9 overexpression level in transgenic plants with *AtMC4*pro::*AtMC9* in the *atmc4‐1* background.
**Figure S13.** Working model for *At*MC4 as a key calcium signal transducer in wounding responses of leaf tissues.

## Data Availability

The data that support the findings of this study are openly available in NCBI at https://www.ncbi.nlm.nih.gov/nucleotide/, reference number BioProject: PRJNA1095498.
