# Supplementary material for: Plant metacaspases orchestrate wound‐induced pathways for immunity and tissue regeneration
Source: Plant J. 2025 Nov 19;124(4):e70531. doi: 10.1111/tpj.70531 (PMC12629630; doi:10.1111/tpj.70531)
Supplement: Supplementary file 1 — Notes S1. Pertinent information related to type II metacaspases in plants. Methods S1. Additional Materials and Method Details. [file TPJ-124-0-s001.pdf]

Supplementary Materials for

Two plant metacaspases orchestrate wound-induced pathways for immunity and tissue regeneration

Zhili Pang, Haijiao Liu, Qun Liu* and Eric Lam*

*Authors for correspondence: eric.lam@rutgers.edu, qunliu@bnl.gov

Article acceptance date: 06 October, 2025

Notes S1. Pertinent information related to type II metacaspases in plants.

Complex interaction network between two divergent type II metacaspases and their targets. Metacaspases (MCs) are cysteine proteases that were first discovered about 25 years ago through structure-based searches for proteases with homologies to metazoan caspases (Cysteine-dependent Asp-ases), and they were found conserved in plant, fungal and protozoan phyla (Uren et al., 2000). The broadly conserved type I MCs contain structurally similar p20 and p10 domains to those found in caspases and typically have N-terminal domains that can mediate protein-protein interaction. In the green lineage from single-celled algae to angiosperms, an additional type II subtype of MCs with distinct structural motifs is also present (Tsiatsiani et al., 2011). Over the past two decades, while much has been revealed about the structure and biochemical activities of these type II MCs, their key biological roles and pathways of influence remain obscure. A major advance in elucidating the functional role of type II MCs was the discovery that *At*MC9, an acid-activated, calcium-independent type II MC (Fortin & Lam, 2018; Vercammen et al., 2004), is a key convertase for the propeptide Grim Reaper (GRI) to generate the active 11-residue cell death elicitor GRIp (Wrzaczek et al., 2015). This led to speculation that the calcium dependent *At*MC4 may be similarly a convertase for the well-studied Propep1 protein to produce the iDAMP Pep1 (Bartels & Boller, 2015), which was demonstrated and extended in a subsequent study (Hander et al., 2019). The matured 22-residue peptide elicitor Pep1, together with its other 7 members in the *Arabidopsis* Propep family, has been studied for more than 15 years (Huffaker, 2015) and is a model for an iDAMP that activates basal immunity upon abiotic and biotic stresses including

herbivore grazing and fungal attack (Ge et al., 2022). We note the finding that type II MCs are in fact convertases for phytocytokines has a striking similarity to the early known function for Caspase-1 in mammals (Denes et al., 2012) as an interleukin-1 (IL-1) converting enzyme (aka ICE, its original name). IL-1 is a key cytokine in mammals that mediates the inflammatory response during tissue damage and microbial defense, precisely in the same context as iDAMP and MAMP responses in plants.

One of the first discovered families of phytocytokines are the eight-membered Pep elicitor peptides from Arabidopsis, which are conserved in both monocots and eudicots (Bartels et al., 2013; Huffaker, 2015). Expression of these propeptides can be induced by herbivores and mechanical wounding, in addition to activation via MAMPs (Ge et al., 2022). Once processed to the mature 24 residue peptide, Pep1 can bind to the extracellular domain of its RLKs called PEPR1/2 and mediate their rapid activation by heterodimerization with the adaptor RLK partner BAK1 (BRASINOSTERIOD INSENSITIVE-ASSOCIATED KINASE 1), which then initiate a phosphorylation cascade involving BIK1 and PBL to mobilize basal immunity functions (Schulze et al., 2010; Yamada et al., 2016). However, the key protease and cellular events that are needed to orchestrate the key step of Propep1 cleavage were only revealed in 2019 (Hander et al., 2019). A highly conserved Ca^{2+}-dependent protease in plants (Fortin & Lam, 2018; Watanabe & Lam, 2011b), *At*MC4 from the type II metacaspase family was found to play a key role in proteolytic processing of Propep1 upon mechanical wounding of *Arabidopsis* leaf tissues (Hander et al., 2019). Loss-of-function *atmc4* mutant plants do not show detectable cleavage of Propep1 upon wounding of leaf tissues, while laser ablation to generate cell-specific damage was used to correlate rapid activation of calcium fluxes upon cell damage and localized activation of Propep1 cleavage by *At*MC4 in the cytoplasm (Hander et al., 2019). Thus, the rapid and localized activation of *At*MC4 to produce Pep1 has the properties to be one of the primary events in the perception and response to cellular damage (Hoermayer & Friml, 2019). Together with the convertase function of *At*MC9 for GRIp (Wrzaczek et al., 2015), these results suggest that this family of conserved plant proteases could act as propeptide maturases to generate various types of phytocytokines.

Aside from *At*MC4 and *At*MC9, there are four other type II MCs in Arabidopsis. However, these other four genes have much lower transcript levels under ambient conditions in diverse tissues of *Arabidopsis* with more significant levels found in roots (Lam & Zhang, 2012;

Tsiatsiani et al., 2013). In the work by Hander et al. (2019), loss of *At*MC4 results in a dramatic absence of Propep1 processing activities in leaves upon compression wounding with forceps, while some Propep1 processing activities remain in the root upon similar wounding treatment. This result indicates that *At*MC4 is critical for Propep1 processing to generate Pep1 elicitor in leaves upon mechanical damage, while one or more of the other five type II MCs may be active in the root tissues for Pep1 release. Using laser ablation with transgenic plants expressing tagged Propep1, the rapid cleavage of the tonoplast surface attached Propep1 can be tracked as well as the tagged Pep1 that was liberated into the cytosol, shortly after the rapid influx of extracellular calcium into the cell was initiated by disruption of cell wall and membrane integrity. Thus, calcium mobilization upon wounding can lead to rapid and localized autolytic activation of *At*MC4, which requires millimolar levels of calcium (Hander et al., 2019; Watanabe & Lam, 2011b), and its cleavage of target substrates in the cytosol. Currently, however, there are few validated substrates for *At*MC4 and other type II metacaspases with demonstrated *in vivo* functions aside from Propep1 and GRI. To overcome this bottleneck, we leverage the protein structure prediction algorithm AlphaFold2 (Jumper et al., 2021) to test whether differential preference exists among the 6 type II MCs toward the eight members of the Propep family.

In this work, we also present our approach using comparative transcriptomics to examine how wild-type (WT) and MC mutant plants may differ in their global transcript landscape upon mechanical wounding of Arabidopsis leaves, using the wounding protocol described previously (Hander et al., 2019). While we expect some of the genes in the basal immune response pathway that are activated by Pep1 would be suppressed in *atmc4* plants, it is unknown what other wound-responsive genes may depend on *At*MC4. It is also unknown in terms of the extent of overlap between genes inducible through the Propep1-*At*MC4-Pep1 module during wounding and those induced by flg22, a well-characterized MAMP elicitor with robust induction of basal immunity to many microbes while distinct from wounding (Ge et al., 2022; Rhodes et al., 2021). In addition, we also studied loss-of-function mutant in *atmc9*, a distinct type II MC that is independent of calcium but instead requires an acidic pH of 5.5 for activation (Fortin & Lam, 2018; Liu et al., 2025; Vercammen et al., 2004). While *At*MC9 was found to be a key protease to process the propeptide for GRIp to an 11-residue peptide that can activate programmed cell death upon oxidative stress, its role in basal immunity induction has not been characterized and

whether it participates in the wounding response has not been reported. Our studies thus explore how iDAMP signals integrate with, while also distinct from, MAMP-dependent pathways.

Methods S1 Additional Materials and Method Details

Structure-based approach to the identification of potential substrate targets for type II metacaspases. In searching for a method to identify potential protein targets of metacaspases, we tested if AlphaFold2 (Jumper et al., 2021), an AI-driven protein structure prediction algorithm, can be used to predict protein-protein interactions (PPIs) between *At*MC4 and substrate candidates. Based on our high-resolution crystal structures (Zhu et al., 2020), under low calcium concentrations *At*MC4 is in a zymogen state and its active site is blocked by Lys225 from its linker region. To ensure the enzyme can be in its activated state and competent for substrate interaction, we removed the linker (residues 183-325) from its sequence for prediction of its structure as a complex with potential substrates. All predictions were performed using three workstations with a total of six Nvidia RTX3090 GPUs. For our structure-based screen, we deployed three filters to identify potential *At*MC4 targets. First, we utilize the clash score calculated by PHENIX (Liebschner et al., 2019) to remove candidates that have more than 50 clashes with *At*MC4. Second, we calculated the PPI interface area using the CCP4 program PISA (https://www.ccp4.ac.uk/) and used a cutoff of 500 $Å^2$ to remove loosely bound proteins. Third, we measured the distance between the catalytic residue C139 thiol group (SG) in *At*MC4 and the closest protein atom and removed targets which are more than 4.5 Å away. Since *At*MC4 cleaves its target protein after a lysine or arginine residue at the P1 position, as a further refinement our analysis prioritized a shorter distance between the C139 SG and arginine/lysine carbonyl oxygen of the target substrates as optimal. Leveraging Coot (Emsley & Cowtan, 2004) for detailed visual examination, we conducted pairwise analyses between the six type II MCs from Arabidopsis with the eight known *Arabidopsis* Propep to reveal any differential substrate preferences between type II MCs. Using the same approach as we have described above for *At*MC4, the linker domain for each type II MCs was removed informatically and then tested for potential interaction with each of the 8 Propep sequences, applying the same three filters as we described.

Plant materials and growth conditions. *Arabidopsis thaliana* accession Columbia (Col-0) and its mutants *atmc4-1* (SAIL_856_D05) and *atmc9-1* (SALK_075814) were used in this study. The

mutant lines *atmc4-1* and *atmc9-1* were originally obtained from the ABRC. Homozygous mutants were selected at the F2 stage and propagated for multiple generations after validation by gDNA PCR with the appropriate primers. The *AtMC4*pro::*AtMC4* vector was constructed by first inserting the *AtMC4* cDNA fragment into the BamHI and SacI double-digested lab vector pNW203 (based on pBI101). A 1.5 kb promoter fragment (XhoI/BamHI) from the *AtMC4* locus (Watanabe & Lam, 2011a) was then inserted into the SalI/BamHI sites in the second cloning step to complete the cassette. Then the constructed vector was mobilized into the *Agrobacterium* strain GV3101 and used for *Arabidopsis* transformation by the floral dipping method and the transgenic plants selected on Kanamycin plates as described previously (Watanabe & Lam, 2011a). Homozygous transgenic lines were obtained by the T2 stage via plate selection followed by validation using gDNA PCR. RT-qPCR was performed to compare the *AtMC4* expression at the RNA level to that in WT or mutant plant lines.

For plant growth, seeds were sterilized with 25% regular bleach (Clorox), germinated on ½ X Murashige and Skoog or ½ X Murashige and Skoog w/o sucrose plates and then stratified at 4°C for 1-3 days. Plates were then moved to a growth chamber (22°C, 8h light/16h dark cycle). For adult plants, one-week-old seedlings were transferred to soil and grown for 3 additional weeks before use.

Overexpression, purification of recombinant proteins. The overexpression of various recombinant proteins used in this work was performed following the protocol previously described (Fortin & Lam, 2018; Zhu et al., 2020). Briefly, after the cell culture reached OD_{600} of 0.4-0.6, IPTG was added to a final concentration of 0.2 mM to induce protein production at 16°C for 20 h. Then cells were collected by centrifugation and resuspended in extraction buffer that contains 25 mM Tris, pH 7.6, 150 mM NaCl, 10 mM DTT, 5% glycerol and Pierce™ Protease Inhibitor Mini Tablets, EDTA-free (ThermoFisher Scientific, REF No. A32955), at 4°C. After cells were lysed, spun down and the supernatant was used to obtain purified GST-Propep1 or Propep3-GST protein using Glutathione Agarose beads from Pierce™ (ThermoFisher Scientific) following the manufacturer's procedure for purification of GST-tagged proteins by the batch method. For preparation of Propep1 without the GST fusion partner to carry out in vivo studies, TEV protease with a 6*His-tag (New England Biolabs) was used for the removal of GST tags from the purified

GST-Propep1 protein. Then the remaining TEV protease was removed by Ni-NTA Agarose resins (Invitrogen by Life Technologies).

rAtMC4 and rAtMC9 protein expression and purification. Plasmids p*ET23a-AtMC4* and p*ET23a-AtMC9* were transformed into Rosetta™ 2(DE3) *pLysS* cells for the production and purification of the corresponding recombinant proteins, as described previously (Fortin & Lam, 2018). Recombinant protein expression was induced with 0.4 mM IPTG at 22°C for 4 - 6 hours, when cells were grown to OD600 between 0.4-0.6. Ni-NTA Agarose resins were used for protein purification. The purified samples were further desalted by using an Amicon® Ultra-0.5 Centrifugal Filter (10 kDa MWCO). Proteins were then resuspended in 50 mM HEPES, pH 7.5, 100 mM NaCl, 1 mM ethylenediaminetetraacetic acid (EDTA), 1 mM dithiothreitol (DTT) and 10% (v/v) glycerol before stored at -80°C until use.

***In vitro* cleavage assay and immunoblotting.** 1 µM of r*At*MC4 was incubated with 0.5 µM of GST-PROPEP1 for 0, 5, 10, 30 min at 30°C in reaction buffer (50 mM HEPES, pH 7.5, 150 mM NaCl, 10% (w/v) glycerol, 5 mM $CaCl_2$, and 10 mM DTT), respectively. For rAtMC9 *in vitro* cleavage assay, 100 mM 2-(*N*-morpholino)-ethanesulfonic acid (MES; pH 5.7), 150 mM NaCl, 10% (w/v) glycerol, and 10 mM DTT were used for reaction buffer. Aliquots of a 3X SDS loading buffer (0.5 M Tris, pH 6.8, 15% (w/v) glycerol, 0.3 M DTT, 5% (w/v) SDS, and bromophenol blue) were added to the samples immediately after incubation and preheated at 70°C for SDS-PAGE analysis. Wet western blotting analysis was performed on Odyssey® nitrocellulose membranes (LI-COR) and incubated with GST-tag polyclonal antibody (Invitrogen, # A-5800). Immunoblots were detected by using IRDye® 800CW Goat anti-Rabbit IgG Secondary Antibody (LI-COR) and imaged by LiCor Odyssey CLx IR Imager (LI-COR).

Leaves treated with wounding or infiltration of synthetic peptides *in planta*. Leaves of 4-week-old Arabidopsis plants from different genetic backgrounds of WT, *atmc4-1*, and *atmc9-1* were used to perform wounding, water infiltration, or peptide treatments, respectively. For Propep1 + wounding treatments, 3 plants were used for one treatment. Two leaves from each plant were wounded by forceps. After the forceps treatment for 2 hours, these leaves were infiltrated with 100 nM Propep1. After another two hours, the treated leaves were collected, and flash frozen in liquid

nitrogen immediately for storage at -80°C until use. For compression wounding, water infiltration, or synthetic peptides (Pep1 or flg22) treatments, the leaves at the same age were wounded by squeezing 5 times with serrated forceps, or infiltrated using a syringe with water only, or 100 nM Pep1 (Synthesized by Biomatik, Purity: 85.49%) or 100 nM flg22 (Synthesized by Genscript, Purity: 97.1%) dissolved in water, respectively. After 4 hours, samples were collected and frozen as described above. The experiment was repeated twice with similar results.

Total RNA extraction. The mirVana™ RNA Isolation kit (Invitrogen) was used to carry out total RNA extraction from the collected leaf samples, following the manufacturer's guidelines. The isolated RNA sample purity was detected by Agarose Gel Electrophoresis and Nanodrop. For Transcriptome analysis, the sample integrity was also analyzed using Agilent 2100 Bioanalyzer by Novogene Ltd.

Transcriptome analysis. Quality checked RNA samples were sequenced on Novaseq 6000 using S4 Flowcell by Novogene Ltd. Non-directional library construction was performed as described (Parkhomchuk et al., 2009). Briefly, poly-T oligo-attached magnetic beads were used for mRNA purification from total RNA. First strand cDNA was synthesized using random hexamer primers, followed by second strand cDNA synthesis using dTTP. Then end repair, A-tailing, adapter ligation, size selection, amplification, and purification were carried out. Illumina Novaseq6000 S4 platform was used for sequencing. FeatureCounts (Liao et al., 2014) v1.5.0-p3 was used to count the read numbers mapped to each gene. Then FPKM (expected number of Fragments Per Kilobase of transcript sequence per Million basepairs sequence) for each gene was calculated based on the length of the gene and reads counts mapped to this gene.

Bioinformatic analysis. Raw data (raw reads) in .fastq format was submitted to Sequence Read Archive (SRA) (https://www.ncbi.nlm.nih.gov/sra). Differentially expressed genes analysis between treatments and no treatment was performed by Novogene Ltd by using DESeq2 (Love et al., 2014). *P* values were adjusted using the Benjamini & Hochberg method. Absolute log2-fold change of 2 and a false discovery rate (FDR) of 0.05 were initially set as the thresholds for significant and high confidence differential expression. Venn Diagram analysis was performed by using Venny 2.1 (Oliveros, 2007-2015). Cluster analysis was performed by using Gene Ontology

(GO) Term Enrichment analysis and was carried out by using the tool: https://www.arabidopsis.org/tools/go_term_enrichment.jsp. The test type was chosen as Fisher's Exact test, and the correction was chosen as Calculate False Discovery Rate (FDR).

Curation of the transcriptome profiling datasets. With the aim of revealing the subset of wound-induced genes that are regulated by *At*MC4 and *At*MC9 through their activity as Propep1 convertases, we carried out a four-way Venn diagram analysis (Figure S6) between the following groups of DEGs: 269 genes of the MC4-dependent DEGs from infiltration wounding with water alone (Figure 3A); 510 genes of the MC4/9-independent, Pep1 infiltrated group (Figure 3B); 452 genes of the MC4-dependent, Propep1+wounding group (Figure 3C); and 44 genes from the MC9-dependent, Propep1+wounding group (Figure 3C). This analysis resulted in the identification of 156 genes by Pep1 that are also induced in an *At*MC4-dependent manner upon water infiltration alone, as well as when treated with PROPEP1+wounding (Figure S6A) and translate to about 37% of the top infiltration-induced genes (Log2-FC >3, Padj <0.05) are regulated through a Propep1-*At*MC4-Pep1 signaling module. However, we note that there are 95 DEGs which are common between the Pep1 infiltrated group and the Propep1+wounding group. The absence of these DEGs to those in the water infiltration group indicates that some of these DEGs may not be induced by pressure infiltration alone but they could be induced under compression wounding and are Pep1 responsive while *At*MC4-dependent. To test this, we further compared the Pep1 induced DEGs with the wounding treatment groups of *At*MC4-dependent (231 DEGs) and *At*MC4/9 co-dependent (141 DEGs). This identified 15 and 5 additional wound-induced DEGs, respectively from the two wounding groups, that are regulated by *At*MC4, and also Pep1-induced from the group of 95 DEGs that are not found with water infiltration alone (Figure S6B). From these results, we suspect that the converse could also be possible, namely that there are infiltration-specific genes that are not induced by compression wounding. Upon comparison of the wounding-induced DEGs that are regulated by *At*MC4 with our set of 156 Pep1-responsive DEGs from the infiltration treatment, 48 of these turned out to be infiltration specific (Figure S7). Together with the 20 DEGs that were recovered from the analysis of the group of 95 DEGs discussed above (Figure S6B), we estimated there are 128 out of the 377 top induced genes (Log2-FC > 3) upon compression wounding that are mediated through the Propep1-*At*MC4-PEP1 module.

Discussion

Complex roles of *At*MC4 and *At*MC9 as activators and repressors in distinct pathways downstream of the initial tissue damage perception

Based on the biochemical properties for type II MCs that we have previously reported on, the calcium-activated *At*MC4 in the cytosol will be expected to be auto-deactivated after about 15 minutes from the initial calcium spike (Fortin & Lam, 2018; Watanabe & Lam, 2011b), further production of Pep1 through the action of *At*MC9 that may be activated by cytosolic acidification in the wounded and neighboring cells (Bosch & Franklin-Tong, 2024) could facilitate systemic propagation of this defense elicitor. This cascade of wound response signaling may be further aided by the secondary induction of other phytocytokines, especially in the PROSCOOP family members (Rhodes et al., 2021), as well as members of the phytosulfokine family (Tost et al., 2021), both of which are known to stimulate defense functions and growth control, respectively, as peptide hormones. It is also intriguing to find that another member of the type II MC family, *At*MC7, is among those wound-induced DEGs that are co-dependent on *At*MC4 and *At*MC9, but independent of Pep1 (Figure S8). Our AlphaFold2 study indicates that *At*MC7 may generate Pep2 and Pep5 from their precursors (Figure 1A). It would be interesting to explore if *At*MC7 also has an increased number of potential targets aside from Propep family members to expand the possible pathways that this signaling cascade can impact.

Our detailed comparison of DEGs affected by synthetic Pep1 and flg22 also revealed the roles for *At*MC4 and *At*MC9 as suppressors to limit the range of gene targets that could be affected by the respective peptide elicitor (Table 2, Figure S5). In each case, Pep1 in *atmc4-1* and flg22 with *atmc9-1* plants, about 400 more DEGs were de-repressed compared to treatments with WT plants by these peptides. These results are in line with the appreciation that a balance of positive and negative functions is needed to provide fine-tuning of the different defense responses such as wounding versus microbial invasion (Yamada et al., 2016; Ge et al., 2022). Like wounding responsive DEGs, treatment with these two peptide elicitors also involves common and peptide-specific DEGs that encode distinct PROSCOOPs as well as other plant peptide hormones including Propep2, Propep3, CLEs and PSK1. This indicates that these phytocytokines and peptide hormones may have evolved to serve synergistic roles in amplifying wounding and defense

responses. However, a noticeable feature that we observed in ectopic infiltration of the two peptide elicitors is the presence of strongly induced DEGs annotated as non-coding RNAs (ncRNA), which we did not find amongst highly induced, wound-responsive DEGs. While some of these encode microRNAs, many of them appear to be genuine lncRNAs that do not encode peptides or functional small RNAs. One well-studied example is *ELENA1*, first discovered as a 589-nucleotide long lncRNA rapidly induced by Elf18 and flg22 elicitor peptides (Seo et al., 2017). From our results, we confirmed that *ELENA1* is one of the highly induced DEGs found with flg22 treatment, but we found that it is not significantly induced by Pep1 on WT plants. However, with *atmc9-1* plants, *ELENA1* became inducible by Pep1 to a similar extent as with flg22 on WT plants, while at least two uncharacterized lncRNAs (At5G09125 and At2G09925) are similarly de-repressed from Pep1 activation in the *atmc4-1* and *atmc9-1* background, respectively (Table 2). Given the roughly five-fold more DEGs that are affected by ectopic peptide elicitor treatments compared to that found with wounding alone, it is tempting to suggest that the induction of distinct subsets of lncRNAs could play a role to help mediate more dramatic global changes in chromatin organization that can result in the segregation of a large number of genes into transcriptionally active domains (TADs). This could take place via interaction between the enhancers of these genes and distinct lncRNAs bound to specific MED complexes, as has been found for multiple loci in the human genome (Bhat et al., 2021). While this model is consistent with the results from examining the mechanism that *ELENA1* uses to mediate activation of PR-1 by flg22 (Seo et al., 2017), whether increase of *ELENA1* and other lncRNAs also could lead to formation of specific TADs in the Arabidopsis genome remains to be confirmed. In any case, elucidating how *At*MC9 may block induction of *ELENA1* and other lncRNAs by Pep1 signaling should reveal novel insights on the role of metacaspases as modulators of global transcriptional control.

Supplemental Figures (Attached)

Figure S1. Supporting data for confirmation of the predicted cleavage and target sequence of Propep3 by *At*MC9, but not *At*MC4.

Figure S2. Workflow and experimental design for identification of metacaspase-dependent genes via wounding and Pep1 signaling.

Figure S3. *At*MC4 is also a key mediator for transcriptional repression upon wounding in leaves.

Figure S4. Heatmap analysis shows similar transcriptional response to two physical treatments in *Arabidopsis thaliana* leaf tissues.

Figure S5. Summary of genes induced (log2-fold change >2, padj <0.05) by various treatments in 3 genetic backgrounds.

Figure S6. Overlap of Pep1 induced DEGs with MC4-dependent genes upon infiltration reveal gene set that is activated via a Propep1-*At*MC4-Pep1 signaling module.

Figure S7. Initial curation of wounding-induced DEGs that are modulated by *At*MC4.

Figure S8. Overlap of transcriptional response between infiltration wounding, flg22 and Pep1 treatments in three different genetic backgrounds.

Figure S9. Validation of selected reporter marker genes for four distinct DEG groups using RT-qPCR.

Figure S10. Complementation of *atmc4-1* via transgenic expression of *AtMC4* cDNA under its cognate promoter.

Figure S11. Phenotypes of Root-from-leaf assay in different genetic background.

Figure S12. Verification of the *At*MC9 overexpression level in transgenic plants with *AtMC4*pro::AtMC9 in the *atmc4-1* background.

Figure S13. Working model for *At*MC4 as a key calcium signal transducer in wounding responses of leaf tissues.

Supplemental Tables as a separate Excel Workbook due to additional data too large for a PDF

Table S1. List of primers used in this study.

Table S2. Overrepresented gene ontology (GO) terms for *At*MC4-dependent upregulated genes in wounding leaf tissues compared with no treatment.

Table S3. Overrepresented gene ontology (GO) terms for *At*MC4 and *At*MC9 co-dependent upregulated genes in wounding leaf tissues compared with no treatment.

Table S4. Curated DEGs which are *AtMC4*-dependent and Pep responsive (Pep+).

Table S5. GO analysis of Pep+ DEGs.

Table S6. Curated DEGs which are *AtMC4*-dependent but not Pep1-inducible (Pep-).

Table S7. GO analysis for Pep- DEGs.

Table S8. Combined Pep+ and Pep- GOs sorted for common and specific pathways between the two groups.

Table S9. Annotated functions for transcription factor (TF) encoding DEGs from the Pep+ and Pep- groups.

References

Bartels, S. & Boller, T. (2015). Pep? Plant elicitor peptides at the crossroads of immunity, stress, and development. *Journal of Experimental Botany,* **66**,5183-5193.

Bartels, S., Lori, M., Mbengue, M., Van Verk, M., Klauser, D., Hander, T. *et al.* (2013). The family of Peps and their precursors in Arabidopsis: differential expression and localization but similar induction of pattern-triggered immune responses. *Journal of Experimental Botany,* **64**, 5309-5321.

Bhat, P., Honson, D. & Guttman, M. (2021). Nuclear compartmentalization as a mechanism of quantitative control of gene expression. *Nature Reviews Molecular Cell Biology*, **22**, 653-670.

Bosch, M. & Franklin-Tong, V. (2024). Regulating programmed cell death in plant cells: Intracellular acidification plays a pivotal role together with calcium signaling. *The Plant Cell*, ***36***, 4692-4702.

Denes, A., Lopez-Castejon, G. & Brough, D. (2012). Caspase-1: is IL-1 just the tip of the ICEberg? *Cell Death & Disease*, **3**, e338-e338.

Emsley, P. & Cowtan, K. (2004). Coot: model-building tools for molecular graphics. *Biological crystallography*, **60**, 2126-2132.

Fortin, J. & Lam, E. (2018). Domain swap between two type-II metacaspases defines key elements for their biochemical properties. *The Plant Journal*, **96**, 921-936.

Ge, D., Yeo, I. C. & Shan, L. (2022) Knowing me, knowing you: Self and non-self recognition in plant immunity. *Essays in Biochemistry,* **66**, 447-458.

Hander, T., Fernández-Fernández, Á. D., Kumpf, R. P., Willems, P., Schatowitz, H., Rombaut, D. *et al.* (2019). Damage on plants activates Ca2+-dependent metacaspases for release of immunomodulatory peptides. *Science*, **363**, eaar7486.

Hoermayer, L. & Friml, J. (2019). Targeted cell ablation-based insights into wound healing and restorative patterning. *Current Opinion in Plant Biology,* **52**, 124-130.

Huffaker, A. (2015). Plant elicitor peptides in induced defense against insects. *Current Opinion in Insect Science*, **9**, 44-50.

Jumper, J., Evans, R., Pritzel, A., Green, T., Figurnov, M., Ronneberger, O. *et al.* (2021). Highly accurate protein structure prediction with AlphaFold. *Nature*, **596**, 583-589.

Lam, E. & Zhang, Y. (2012). Regulating the reapers: activating metacaspases for programmed cell death. *Trends in Plant Science*, **17**, 487-494.

Liao, Y., Smyth, G. K. & Shi, W. (2014). FeatureCounts: an efficient general purpose program for assigning sequence reads to genomic features. *Bioinformatics*, **30**, 923-930.

Liebschner, D., Afonine, P. V., Baker, M. L., Bunkóczi, G., Chen, V. B., Croll, T. I. *et al.* (2019). Macromolecular structure determination using X-rays, neutrons and electrons: recent developments in Phenix. *Biological Crystallography*, **75**, 861-877.

Liu, H., Henderson, M., Pang, Z., Zhang, Q., Lam, E. & Liu, Q. (2025). Structural determinants for pH-dependent activation of a plant metacaspase. *Nature Communications*, **16**, 4973.

Love, M. I., Huber, W. & Anders, S. (2014). Moderated estimation of fold change and dispersion for RNA-seq data with DESeq2. *Genome biology*, **15**, 550.

Oliveros, J. C. (2007-2015). Venny. An interactive tool for comparing lists with Venn's diagrams. https://bioinfogp.cnb.csic.es/tools/venny/index.html.

Parkhomchuk, D., Borodina, T., Amstislavskiy, V., Banaru, M., Hallen, L., Krobitsch, S. ***et***

al. (2009). Transcriptome analysis by strand-specific sequencing of complementary DNA. *Nucleic acids research*, **37**, e123-e123.

Rhodes, J., Yang, H., Moussu, S., Boutrot, F., Santiago, J. & Zipfel, C. (2021). Perception of a divergent family of phytocytokines by the Arabidopsis receptor kinase MIK2. *Nature Communications*, **12**, 705.

Schulze, B., Mentzel, T., Jehle, A. K., Mueller, K., Beeler, S., Boller, T. *et al.* (2010). Rapid heteromerization and phosphorylation of ligand-activated plant transmembrane receptors and their associated kinase BAK1. *Journal of Biological Chemistry*, **285**, 9444-9451.

Seo, J. S., Sun, H. X., Park, B. S., Huang, C. H., Yeh, S. D., Jung, C. *et al.* (2017). ELF18-INDUCED LONG-NONCODING RNA associates with mediator to enhance expression of innate immune response genes in Arabidopsis. *The Plant Cell*, **29**, 1024-1038.

Tost, A. S., Kristensen, A., Olsen, L. I., Axelsen, K. B. & Fuglsang, A. T. (2021). The PSY peptide family—expression, modification and physiological implications. *Genes*, **12**, 218.

Tsiatsiani, L., Timmerman, E., De Bock, P. J., Vercammen, D., Stael, S., Van De Cotte, B. *et al.* (2013). The Arabidopsis metacaspase9 degradome. *The Plant Cell*, **25**, 2831-2847.

Tsiatsiani, L., Van Breusegem, F., Gallois, P., Zavialov, A., Lam, E. & Bozhkov, P. V. (2011). Metacaspases. *Cell Death & Differentiation*, 18, 1279-1288.

Uren, A. G., O'Rourke, K., Aravind, L. A., Pisabarro, M. T., Seshagiri, S., Koonin, E. V. *et al.* (2000). Identification of paracaspases and metacaspases: two ancient families of caspase-like proteins, one of which plays a key role in MALT lymphoma. *Molecular Cell*, **6**, 961-967.

Vercammen, D., Van De Cotte, B., De Jaeger, G., Eeckhout, D., Casteels, P., Vandepoele, K. *et al.* (2004). Type II metacaspases Atmc4 and Atmc9 of *Arabidopsis thaliana* cleave substrates after arginine and lysine. *Journal of Biological Chemistry*, **279**, 45329-45336.

Watanabe, N. & Lam, E. (2011a). Arabidopsis metacaspase 2d is a positive mediator of cell death induced during biotic and abiotic stresses. *The Plant Journal*, **66**, 969-982.

Watanabe, N. & Lam, E. (2011b). Calcium-dependent activation and autolysis of *Arabidopsis* metacaspase 2d. *Journal of Biological Chemistry*, **286**, 10027-10040.

Wrzaczek, M., Vainonen, J. P., Stael, S., Tsiatsiani, L., Help-Rinta-Rahko, H., Gauthier, A. *et al.* (2015). GRIM REAPER peptide binds to receptor kinase PRK 5 to trigger cell death in *Arabidopsis*. *The EMBO Journal*, **34**, 55-66.

Yamada, K., Yamashita-Yamada, M., Hirase, T., Fujiwara, T., Tsuda, K., Hiruma, K. & Saijo, Y. (2016). Danger peptide receptor signaling in plants ensures basal immunity upon pathogen-induced depletion of BAK1. *The EMBO Journal,* **35**, 46-61.

Zhu, P., Yu, X. H., Wang, C., Zhang, Q., Liu, W., McSweeney, S. *et al.* (2020). Structural basis for Ca2+-dependent activation of a plant metacaspase. *Nature communications*, **11**, 2249.
