# Supplementary material for: Plant metacaspases orchestrate wound‐induced pathways for immunity and tissue regeneration
Source: Plant J. 2025 Nov 19;124(4):e70531. doi: 10.1111/tpj.70531 (PMC12629630; doi:10.1111/tpj.70531)
Supplement: Supplementary file 3 — Figure S1. Supporting data for confirmation of the predicted cleavage and target sequence of Propep3 by AtMC9, but not AtMC4. Figure S2. Workflow and experimental design for identification of metacaspase‐dependent genes via wounding and Pep1 signaling. Figure S3. AtMC4 is also a key mediator for transcriptional repression upon wounding in leaves. Figure S4. Heatmap analysis shows similar transcriptional response to two physical treatments in Arabidopsis thaliana leaf tissues. Figure S5. Summary of genes induced (log2‐fold change >2, P adj <0.05) by various treatments in three genetic backgrounds. Figure S6. Overlap of Pep1‐induced DEGs with MC4‐dependent genes upon infiltration reveal gene set that is activated via a Propep1‐AtMC4‐Pep1 signaling module. Figure S7. Initial curation of wounding‐induced DEGs that are modulated by AtMC4. Figure S8. Overlap of transcriptional response between infiltration wounding, flg22, and Pep1 treatments in three different genetic backgrounds. Figure S9. Validation of selected reporter marker genes for four distinct DEG groups using RT‐qPCR. Figure S10. Complementation of atmc4‐1 via transgenic expression of AtMC4 cDNA under its cognate promoter. Figure S11. Phenotypes of root‐from‐leaf assay in different genetic background. Figure S12. Verification of the AtMC9 overexpression level in transgenic plants with AtMC4pro::AtMC9 in the atmc4‐1 background. Figure S13. Working model for AtMC4 as a key calcium signal transducer in wounding responses of leaf tissues. [file TPJ-124-0-s002.pdf]

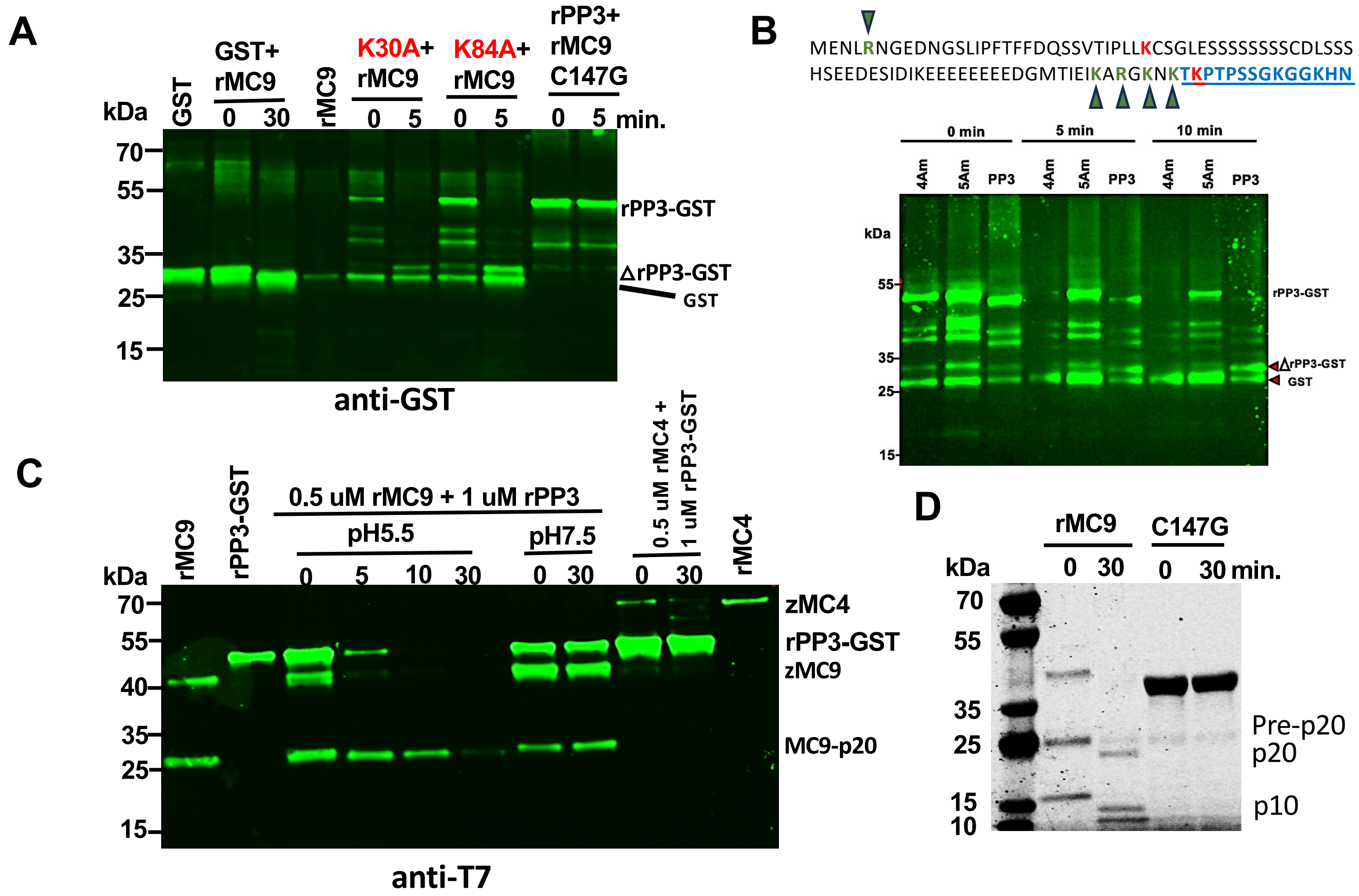

Figure S1. Supporting data for confirmation of the predicted cleavage and target sequence of Propep3 by *At*MC9, but not *At*MC4. (A) GST protein was purified from bacteria with a vector p*ET23a(+)-GST* for GST only. WB detection using anti-GST antibody showed the GST tag is not significantly cleaved by rMC9 protein after 30 min. at pH5.5, 30°C. K30 is a cleavage site predicted by AlphaFold2 while K84 is near the N-terminus of the mature Pep3 sequence reported from previous work. However, the results showed that K30 and K84 are not major cleavage sites of PP3 by rMC9, with both variants rapidly cleaved by rMC9 within 5 minutes and the accumulation of the processed ΔrPP3-GST. rMC9 mutant rMC9/C147G is unable to cleave rPP3-GST, as expected. **(B)** Sequence of PP3 with residues of interest highlighted. Mature Pep3 is in blue fonts and underlined. K30 and K84 are in red fonts. R5 and the four basic residues upstream of the Pep3 sequence are in green fonts with arrowheads. A mutant with all five of the basic residues R5, K76, R78, K80 and K82 were replaced with Ala (**5Am**) was synthesized. From this synthetic gene, the A5 residue is restored to an Arg by site-directed mutagenesis to create the PP3 variant **4Am**. Both mutants do not accumulate the ΔrPP3-GST product. The 5Am variant is significantly inhibited in its cleavage by rMC9. To better resolve the kinetics of degradation, this reaction was carried out at 25°C instead of 30°C. The R5 residue appears to play a role to initiate rapid degradation of rPP3 while the four basic residues are needed for accumulation of mature Pep3. **(C)** WB detection using anti-T7 antibody showed PP3 can be cleaved by rMC9 only at pH 5.5, but not by rMC4 at pH7.5 in the presence of 5 mM $CaCl_2$. The zymogen form of rMC4 and rMC9 are cleaved under their respective conditions of pH7.5 and pH5.5. **(D)** Coomassie Blue stained gels of rMC9 and rMC9/C147G proteins which were incubated at 30°C at 0 and 30 min. to show autolytic cleavage of the WT but not the active site mutant.

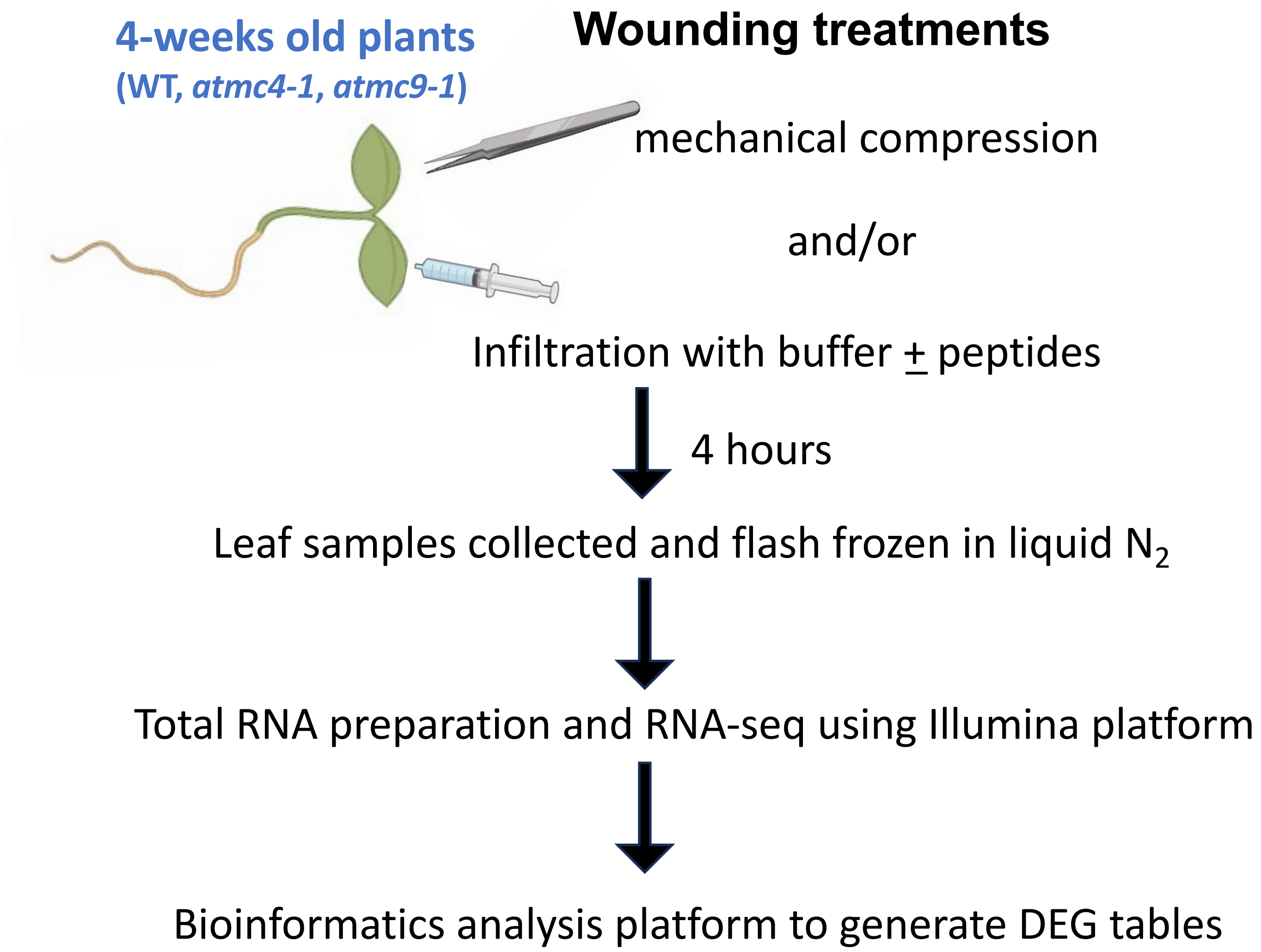

Figure S2. Workflow and experimental design for identification of metacaspase-dependent genes via wounding and Pep1 signaling.

AtMC4 is also a Key Mediator for Transcriptional Repression Upon Wounding in Leaves

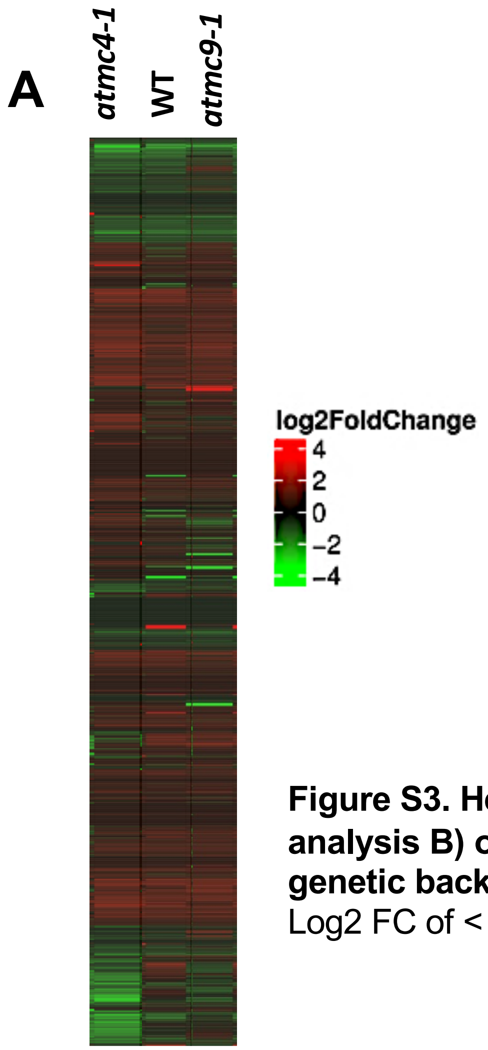

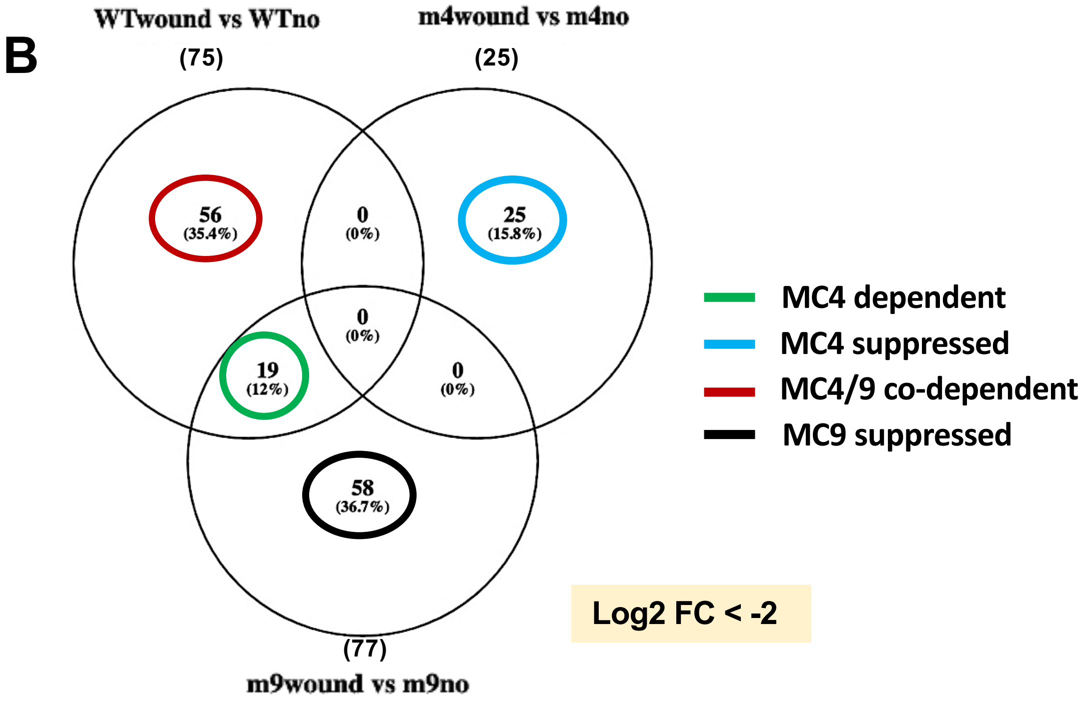

Figure S3. Heat map analysis for global transcript landscape A) and Venn Diagram analysis B) of the difference in wounding-repressed group among three different genetic background. *Arabidopsis thaliana* plants *atmc4-1*, WT, and *atmc9-1* for DEGs with Log2 FC of < -2. The numbers in parentheses are total DEGs in each group.

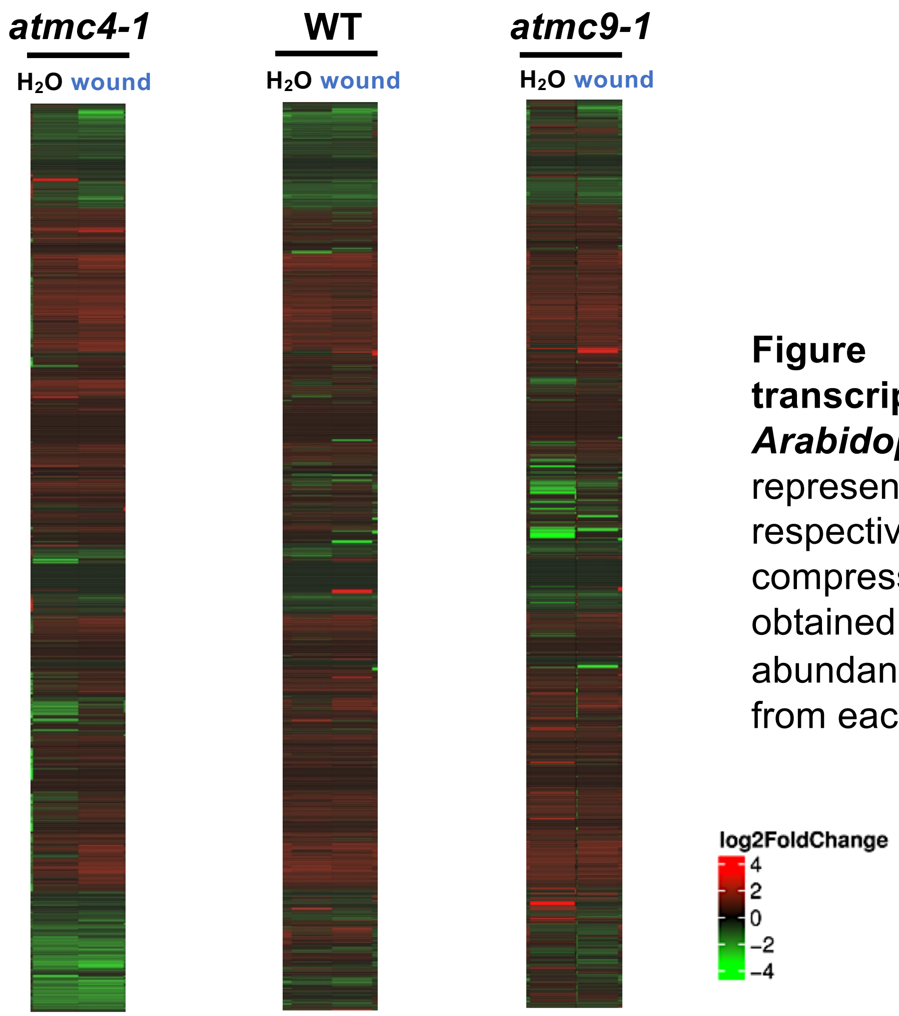

Figure S4. Heatmap analysis shows similar transcriptional response to two physical treatments in *Arabidopsis thaliana* leaf tissues. *Atmc4-1*, WT, *atmc9-1* represents different genetic background plants, respectively. H_2O: water infiltration treatment; Wound: compression by forceps treatment. Log2 FC values were obtained for each gene by comparing the transcript abundance after each treatment to the un-treated controls from each genetic background.

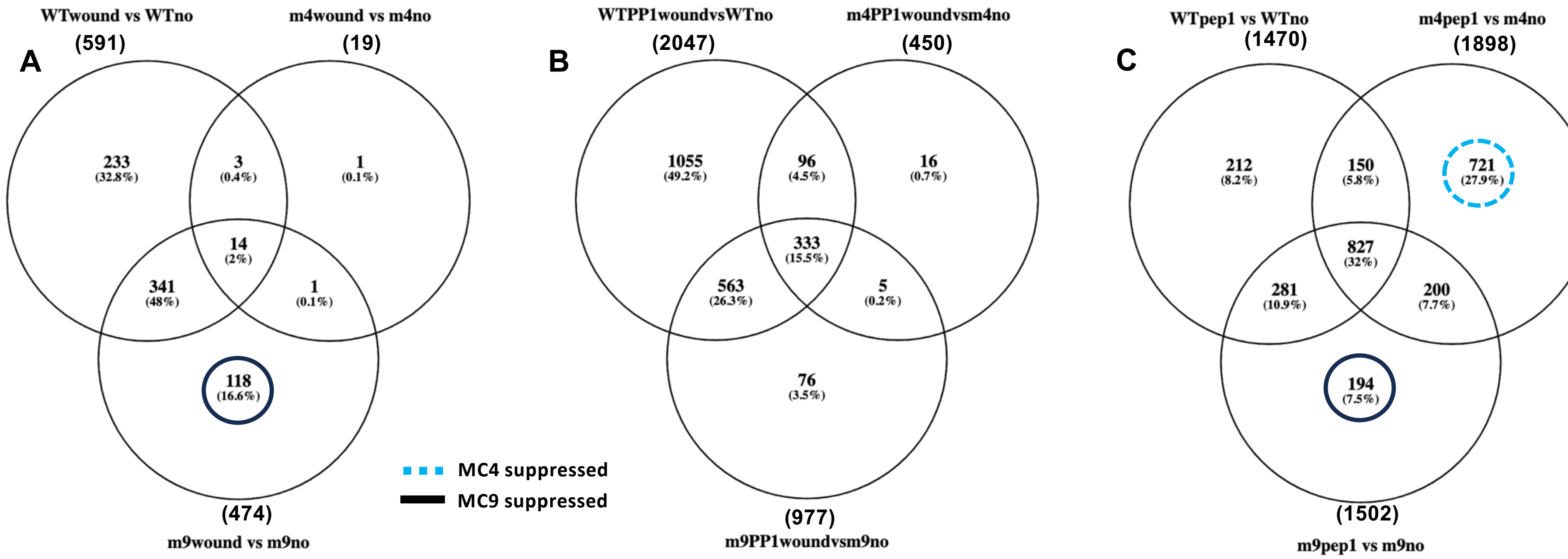

Figure S5. Summary of genes induced (log2-fold change >2, padj <0.05) by various treatments in 3 genetic backgrounds. A) wounding, **B)** Propep1 + wounding**, and C)** Pep1 treatments in WT, *atmc4-1*, and *atmc9-1* of *Arabidopsis thaliana*, respectively. Wound represents wounding treatment by pinching leaves with forceps. After 4 hours, samples were collected for transcriptome analysis. PP1wound represents infiltration leaves with 0.1 μM Propep1 for 2 hours before wounding treatment with forceps for another 2 hours; Pep1 represents infiltration leaves with 0.1 μM Pep1. After 4 hours, samples were collected**.**

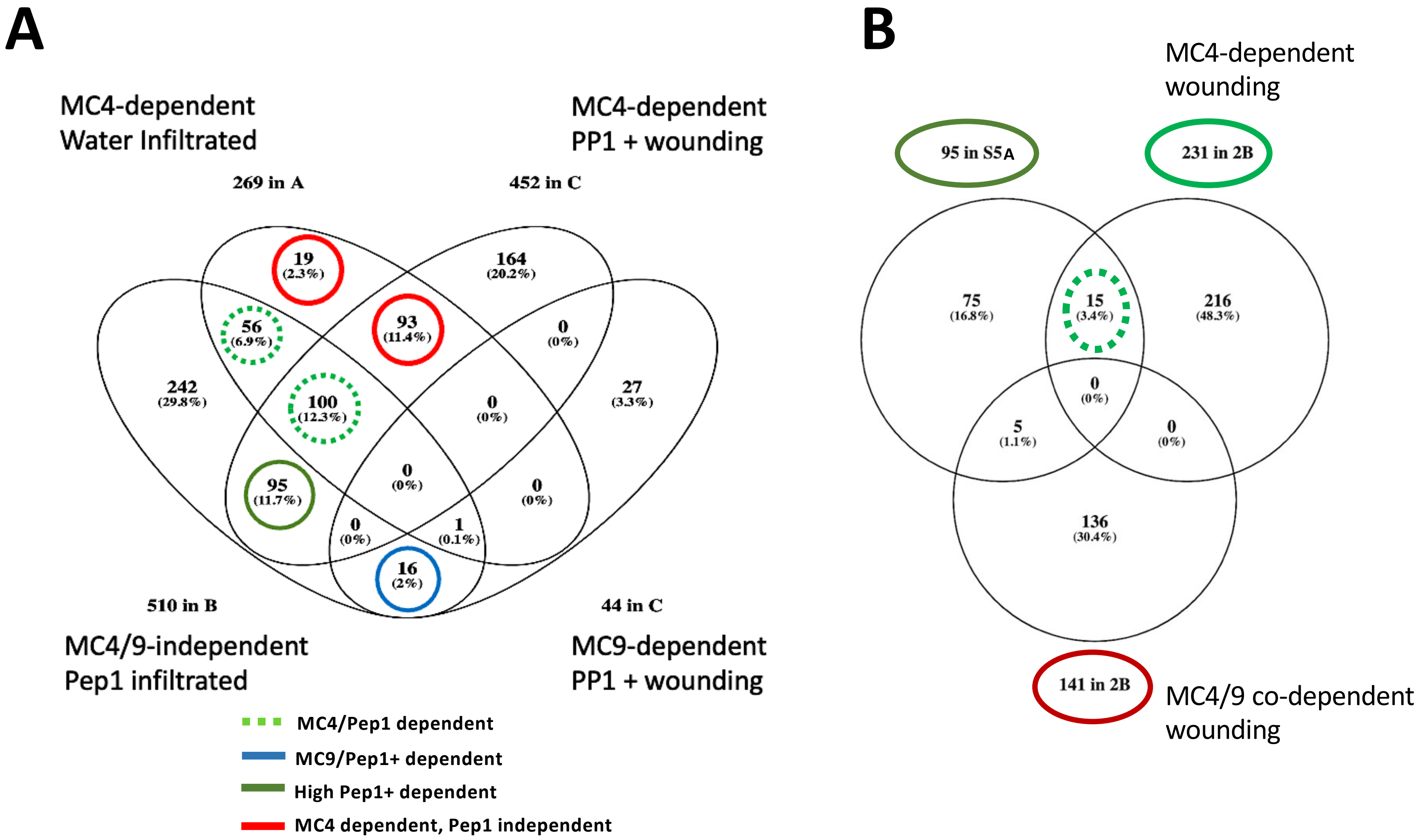

Figure S6. Overlap of Pep1 induced DEGs with MC4-dependent genes upon infiltration reveal gene set that is activated via a Propep1-AtMC4-Pep1 signaling module. A) Overlap of **156** DEGs induced by Pep1 are found out of the **269** total that are activated in an AtMC4-dependent manner upon water infiltration (Fig. 3). In contrast, 16 out of 44 DEGs induced by a combination of Propep1 and additional compression wounding are Pep1 inducible in *atmc9* seedlings. Also, there are **112** MC4-dependent infiltration-induced genes that are not significantly activated by ectopic addition of Pep1. **B)** Curation of additional wound-induced DEGs that are MC4-dependent. Venn diagram analysis of the Pep1-inducible genes from the MC4-dependent and MC4/9 co-dependent DEGs upon compression wounding treatment. 15 additional genes were identified that are not found in the MC4-dependent DEGs from water infiltration treatment (95 DEGs group in A).

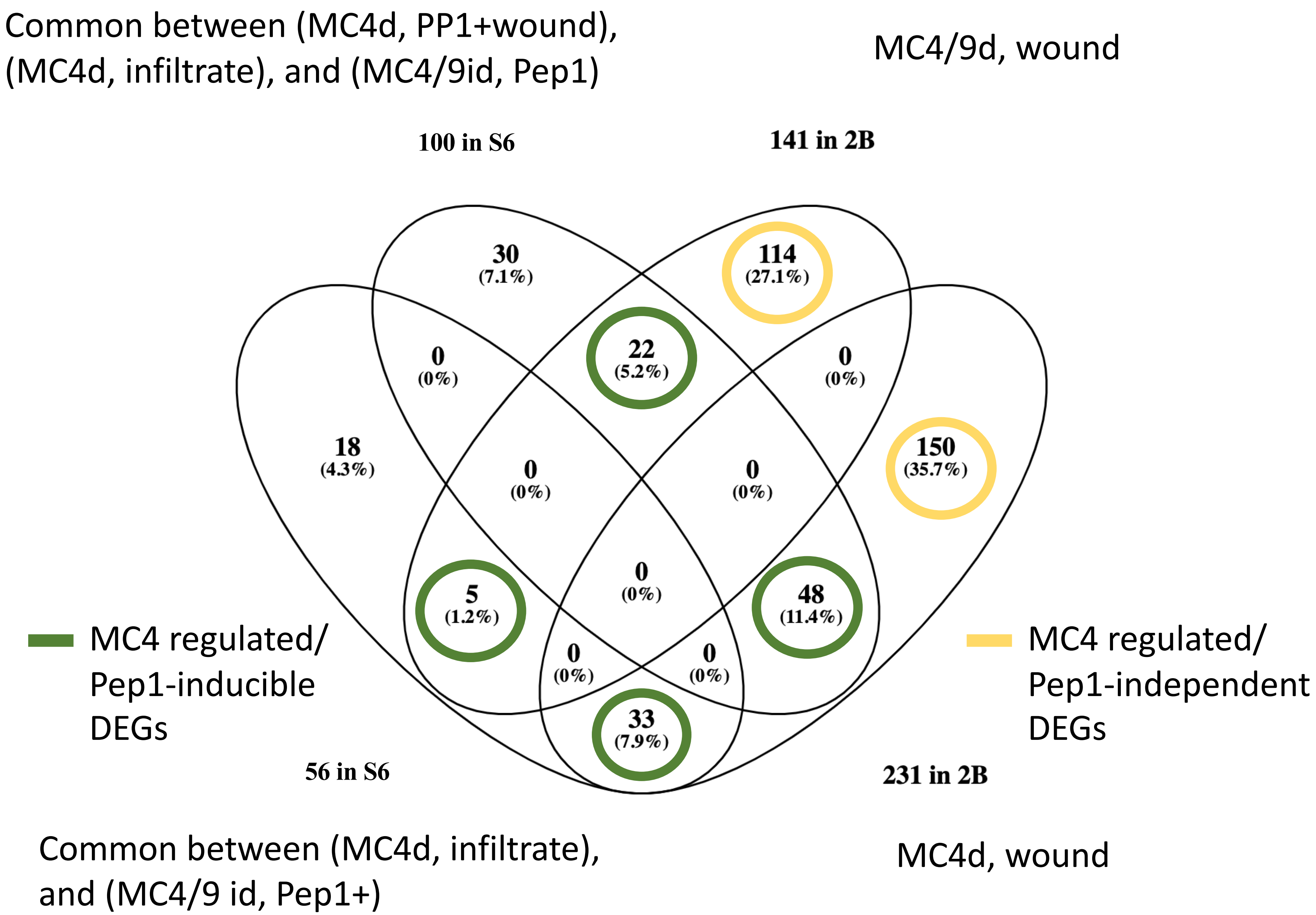

Figure S7. Initial curation of wounding-induced DEGs that are modulated by AtMC4 and Pep1-mediated (green circles) and those that do not involve Pep1 (yellow circles). Selected sets of DEGs curated from fig. S6 and Fig. 2B are used for further Venn analysis. 20 genes are further re-distributed between these two groups due to curation of wounding specific genes from the group of 95 genes in Fig. S6A.

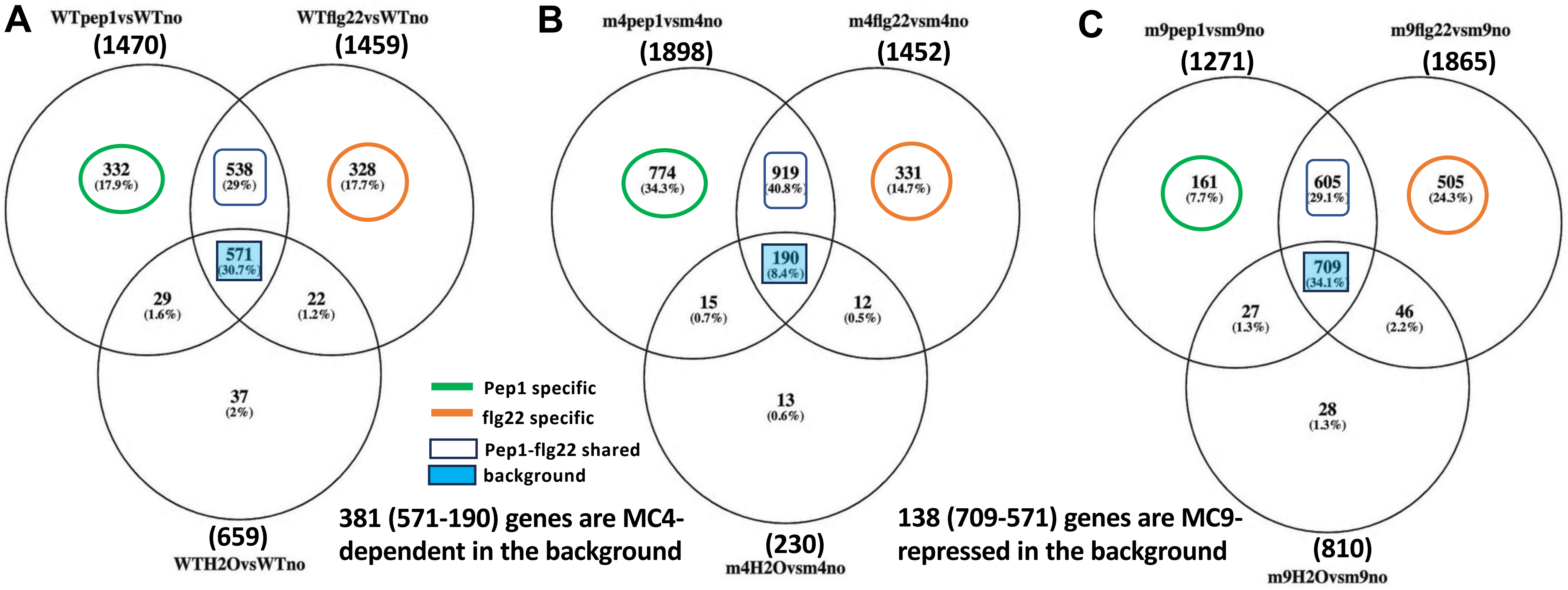

Figure S8. Overlap of transcriptional response between infiltration wounding, flg22 and Pep1 treatments in three different genetic backgrounds. Summary of the overlap between genes induced by water infiltration (H2O), Pep1 in water (pep1), and flg22 peptide in water (flg22) in the genetic background of **A)** wild-type, **B)** *atmc4-1*, and **C)** *atmc9-1* genotypes of *Arabidopsis thaliana*. Differentially expressed genes (DEGs) comparing to no treatment controls from each of the respective genotypes (Log2 FC >2, Padj <0.05) are curated and compared using Venn diagrams. Total number of DEGs in each case is shown in paraentheses above each set of DEGs.The common DEGs between water only and those with the peptides are shown in the central box shown highlighted in blue. The common DEGs between Pep1 and flg22 treatments are shown in the transparent box. The highest number of overlap between the two peptide treatments is found in the *atmc4* background, highlighting the negative function of AtMC4 as repressor of wound induction of defense genes. While the loss of AtMC9 significantly increased the number of genes induced by infiltration wounding and flg22, the number of genes specifically induced by Pep1 decreased (red circles). These results showed that AtMC9 act to repress gene expression induced by wounding and by flg22, while it is required for activation of other genes downstream of Pep1.

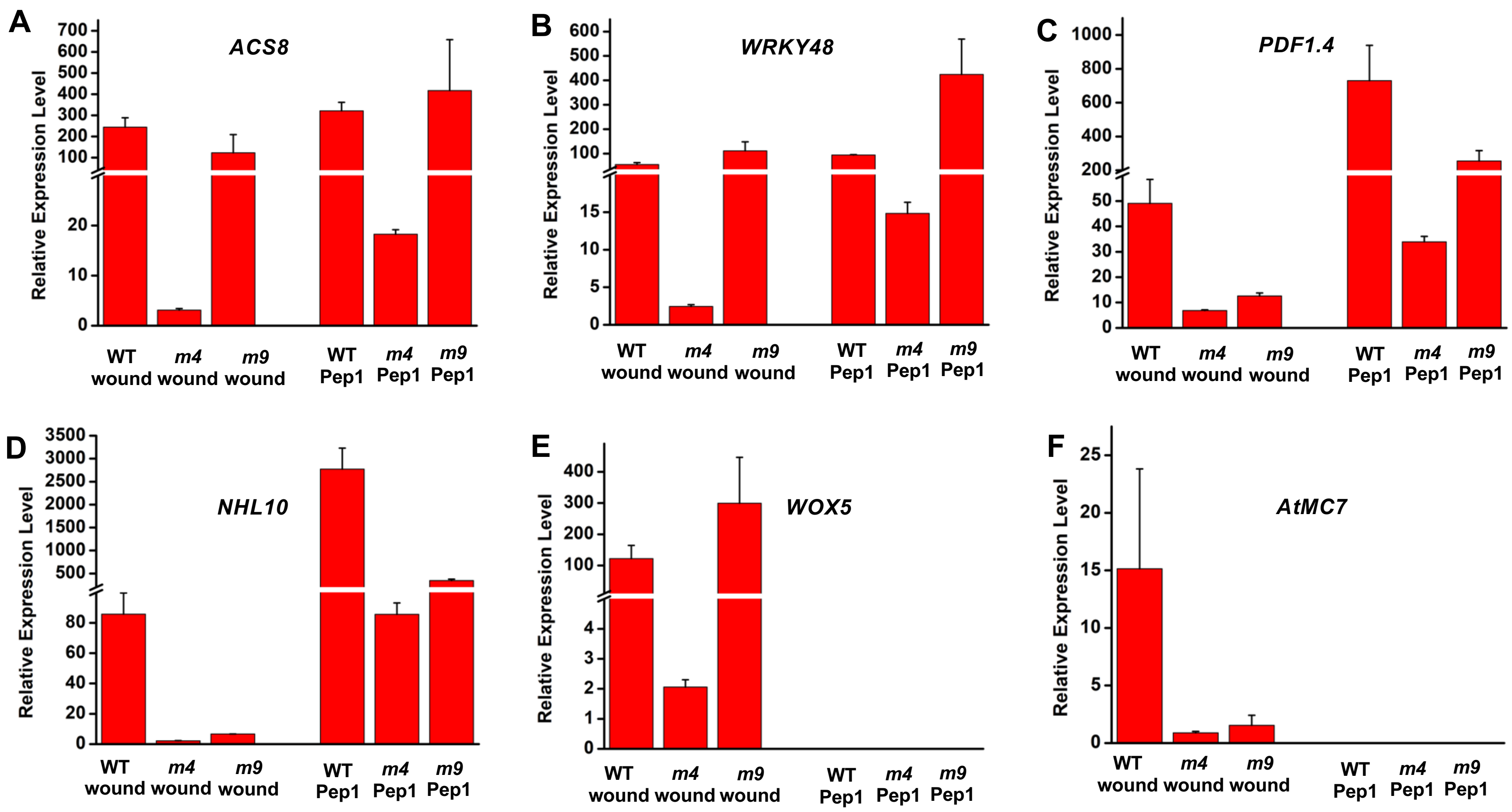

Figure S9. Validation of selected reporter marker genes for four distinct DEG groups using RT-qPCR. The relative expression level for each gene was calculated compared to the no treatment control, respectively. The *UBC21/PEX4* gene was used as reference control. Group 1 genes: *ACS8* **A**), *WRKY48* **B**); Group 2 genes: *PDF1.4* **C**), *NHL10* **D**); Group 3 gene: *WOX5* **E**) ; Group 4 gene: *AtMC7* **F**). Different scales are used for the different genes assayed in order to optimize the resolution of the data for each. Bars show the standard deviation from the mean of three replicates for each assay.

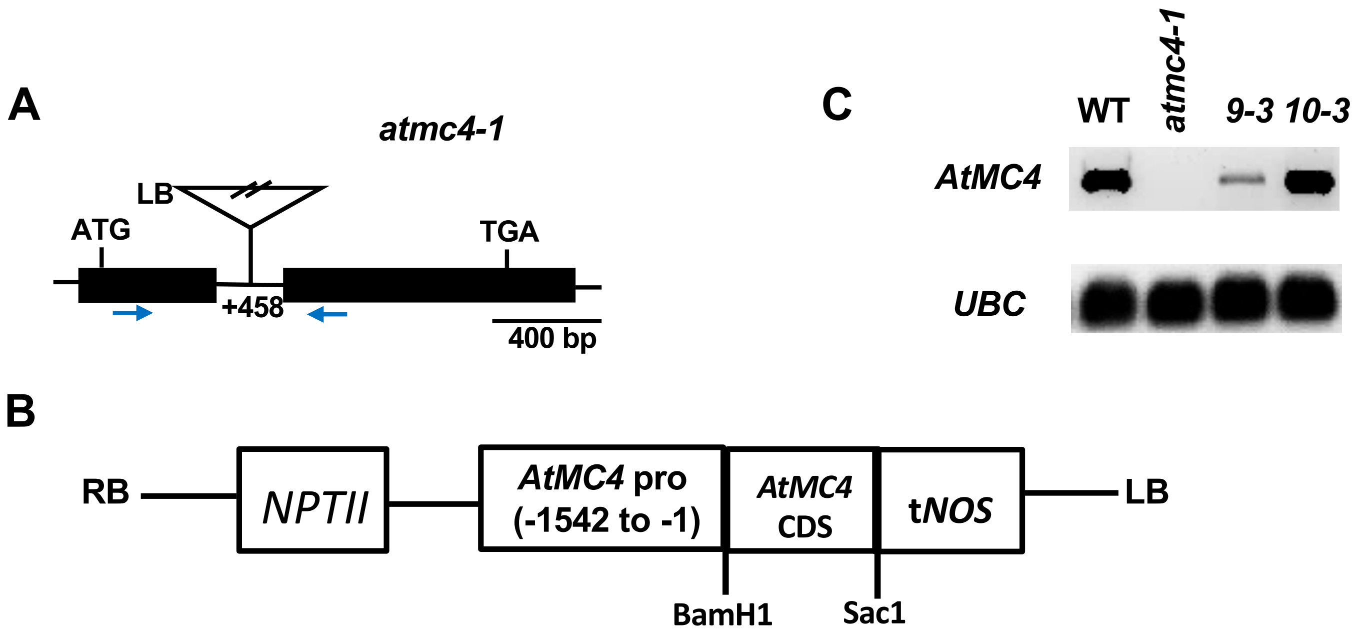

Figure S10. Complementation of *atmc4-1* via transgenic expression of *AtMC4* cDNA under its cognate promoter. A). Structure of the T-DNA insertion locus in *atmc4-1*. **B)** Transgene used for functional complementation. XhoI-BamHI fragment of *AtMC4* promoter (-1542 to -1) was cloned into the SalI-BamHI site of pBI101. BamHI-SacI fragment of *AtMC4* coding sequence (CDS) was cloned to BamHI-SacI site of pBI101. Terminator sequence from the nopaline synthase gene (t*NOS*) of Agrobacterium is downstream of the *AtMC4* cassette. Kanamycin selection is provided by the *NPTII* gene in the vector T-DNA sequence. Size of the boxes are not to scale **C)** Transcript analysis with RT-PCR amplification of *AtMC4* coding sequence from wild-type (WT), *atmc4-1* mutant, and stable transgenic plant lines with *AtMC4*pro::*AtMC4* in the *atmc4-1* genetic background. T3 lines #9-3, and #10-3 for low and high expression were selected for comparison. The *UBC21/PEX4* (At5G25760) gene was used as the reference control. The position of the primers for *AtMC4* gene amplification are shown by the blue arrows in **A**.

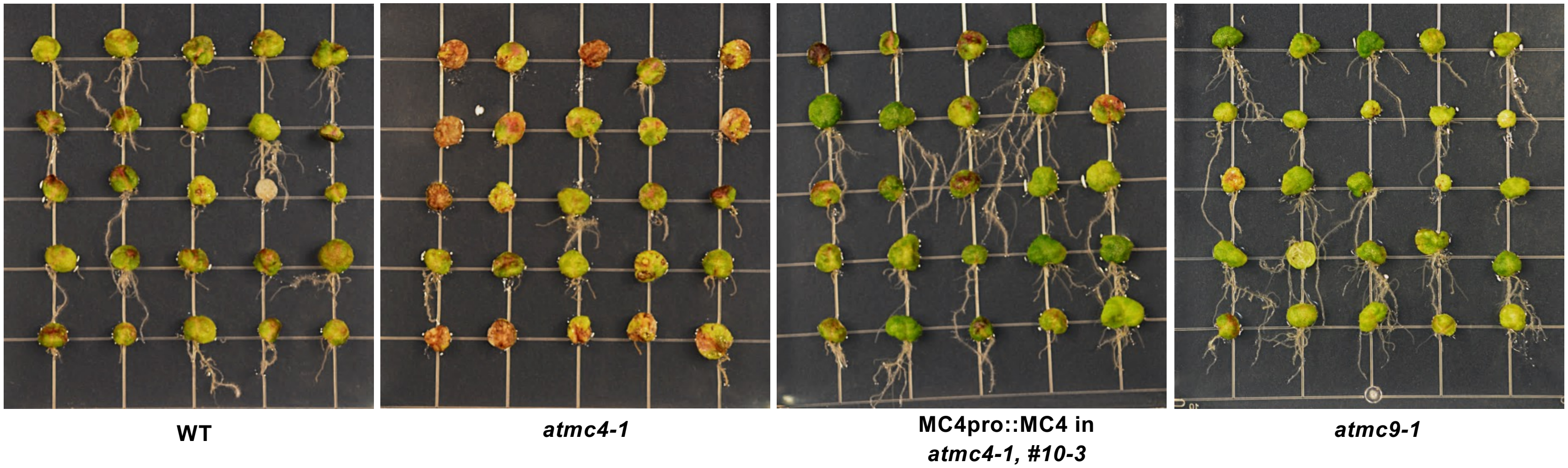

Figure S11. **Phenotypes of Root-from-leaf assay in different genetic background.** Photos of root regeneration frequency assay in different Arabidopsis genetic backgrounds of WT, *atmc4-1*, *AtMC4*pro::*AtMC4* cds in *atmc4-1* #10-3, and *atmc9-1* leaves. Regenerated roots emerging from the cut petiole site is scored after culturing the excised leaves on ½X MS plates in normal day-night cycle as described in Supplemental Information.

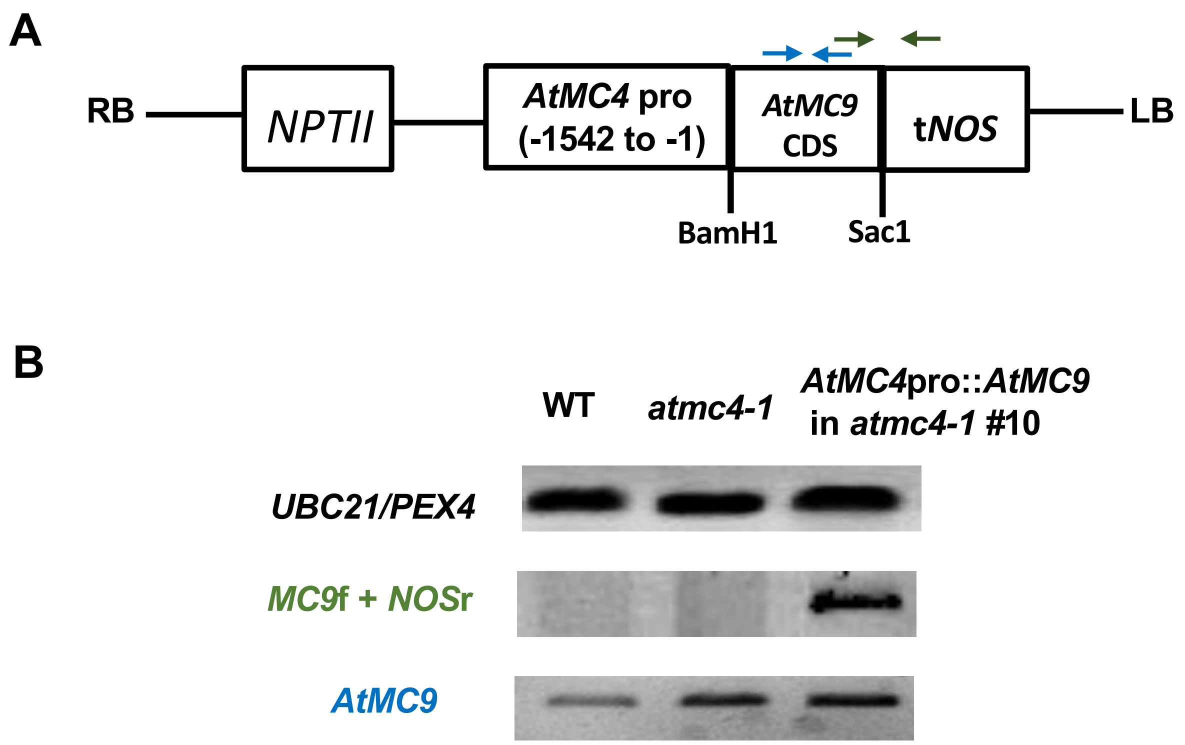

Figure S12. Verification of the AtMC9 overexpression level in transgenic plants *MC4*pro::MC9 in the *atmc4-1* background. A) Transgene used for functional complementation test. An XhoI-BamHI fragment of *AtMC4* promoter (-1542 to -1) was cloned into the SalI-BamHI site of pBI101 vector. BamHI-SacI fragment of *AtMC9* coding sequence (CDS) was cloned to the BamHI-SacI site of pBI101. Terminator sequence from the nopaline synthase gene (t*NOS*) of *Agrobacterium* is downstream of the *AtMC4* promoter expression cassette. Kanamycin selection is provided by the *NPTII* gene in the vector T-DNA sequence. Size of the boxes are not to scale. **B**) RT-PCR analysis of *AtMC9* ectopic expression at the RNA level from transgenic plants with *MC4pro::MC9* in the *atmc4-1* #10 transgenic line. Primers used were indicated by arrows in (A): blue primers are used for the cognate *AtMC9* locus while the green primers are used to detect specifically the transgene locus. *UBC21/PEX4* (At5G25760) expression was used as control for RNA quality and quantity.

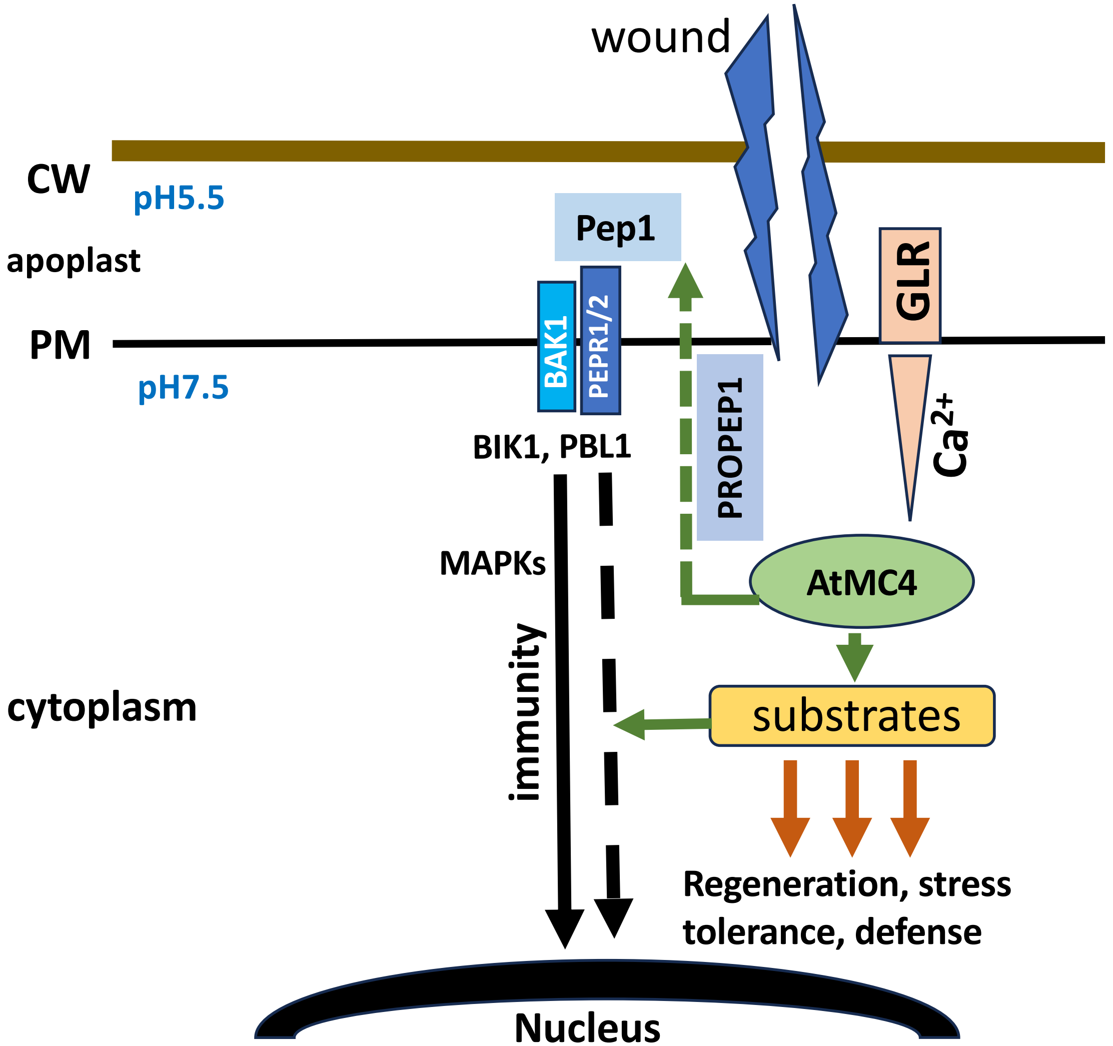

Figure S13. Working model for AtMC4 as a key calcium signal transducer in wounding responses of leaf tissues. GLR: glutamate receptor-like proteins are known to be involved in calcium flux regulation upon wounding in Arabidopsis (Hernandez-Coronado et al., 2022). **Black arrow** indicating basal immunity functions initiated with the cooperation of the adaptor receptor kinase BAK1; **black broken arrow** indicates immunity functions from Pep1 activation that requires additional input by AtMC4 in the cytosol; **green broken arrow** is Propep1 conversion to Pep1 by AtMC4; **green arrows** are cytosolic functions of AtMC4 distinct from Propep1 maturation; **brown arrows** are additional pathways activated by AtMC4 distinct from Pep1-dependent functions.
